# Development and external validation of a clinical prediction model to aid coeliac disease diagnosis in primary care: An observational study

**DOI:** 10.1016/j.eclinm.2022.101376

**Published:** 2022-04-07

**Authors:** Martha M.C. Elwenspoek, Rachel O'Donnell, Joni Jackson, Hazel Everitt, Peter Gillett, Alastair D. Hay, Hayley E. Jones, Gerry Robins, Jessica C. Watson, Sue Mallett, Penny Whiting

**Affiliations:** a The National Institute for Health Research Applied Research Collaboration West (NIHR ARC West), University Hospitals Bristol NHS Foundation Trust, Bristol, BS1 2NT, UK; b Population Health Sciences, Bristol Medical School, University of Bristol, Bristol, BS8 2PS, UK; c Primary Care Research Centre, University of Southampton, Southampton SO16 5ST, UK; d Paediatric Gastroenterology, Hepatology and Nutrition Department, Royal Hospital for Sick Children, Edinburgh EH9 1LF, Scotland, UK; e Department of Gastroenterology, York Teaching Hospital NHS Foundation Trust, York, YO31 8HE, UK; f Centre for Medical Imaging, University College London, 2nd Floor, Charles Bell House, 43-45 Foley Street, London, W1W 7TS, UK

**Keywords:** Coeliac disease, Prediction model, Clinical prediction rule, CPRD

## Abstract

**Background:**

Coeliac disease (CD) affects approximately 1% of the population, although only a fraction of patients are diagnosed. Our objective was to develop diagnostic prediction models to help decide who should be offered testing for CD in primary care.

**Methods:**

Logistic regression models were developed in Clinical Practice Research Datalink (CPRD) GOLD (between Sep 9, 1987 and Apr 4, 2021, n=107,075) and externally validated in CPRD Aurum (between Jan 1, 1995 and Jan 15, 2021, n=227,915), two UK primary care databases, using (and controlling for) 1:4 nested case-control designs. Candidate predictors included symptoms and chronic conditions identified in current guidelines and using a systematic review of the literature. We used elastic-net regression to further refine the models.

**Findings:**

The prediction model included 24, 24, and 21 predictors for children, women, and men, respectively. For children, the strongest predictors were type 1 diabetes, Turner syndrome, IgA deficiency, or first-degree relatives with CD. For women and men, these were anaemia and first-degree relatives. In the development dataset, the models showed good discrimination with a *c*-statistic of 0·84 (95% CI 0·83–0·84) in children, 0·77 (0·77–0·78) in women, and 0·81 (0·81–0·82) in men. External validation discrimination was lower, potentially because ‘first-degree relative’ was not recorded in the dataset used for validation. Model calibration was poor, tending to overestimate CD risk in all three groups in both datasets.

**Interpretation:**

These prediction models could help identify individuals with an increased risk of CD in relatively low prevalence populations such as primary care. Offering a serological test to these patients could increase case finding for CD. However, this involves offering tests to more people than is currently done. Further work is needed in prospective cohorts to refine and confirm the models and assess clinical and cost effectiveness.

**Funding:**

National Institute for Health Research Health Technology Assessment Programme (grant number NIHR129020)

**Research in context**

**Evidence before this study**

MEDLINE, Embase, Cochrane Library, Web of Science, World Health Organization's International Clinical Trials Registry, and the NIH Clinical Trials database were searched (from Jan 1, 1997 until April 16, 2021) using terms for coeliac disease (CD), a prognostic/predictive research filter, physical diseases, signs, and symptoms (based on MeSH, EMTREE), and terms for high risk populations. No prediction models for CD that used symptoms and chronic diseases as predictors were identified.

**Added value of this study**

We developed and validated three models to predict CD in children, women, and men. We identified the following risk factors as important predictors for CD which are not yet mentioned by most guidelines: arthritis, chronic liver disease, delayed puberty, and mood disorders as important predictors for CD in children; fractures, IgA deficiency, and inflammatory bowel disease in women; and cardiovascular disease, chronic liver disease, epilepsy, and psoriasis in both adult men and women.

**Implications of all the available evidence**

The prediction models that we developed are not meant to diagnose CD but could be used to assess whether a patient should be offered a test for CD. If a serological test would be offered to all individuals with at least a 1·5% risk according to the models (equivalent to having any single predictor), only 12% of children, 16% of women, and 13% of men with CD would be missed. Although this would be a substantial improvement compared to current practice, this means offering tests to >55% of people and the cost-effectiveness of this strategy needs to be investigated.

Alt-text: Unlabelled box

## Introduction

Coeliac disease (CD) is one of the most common autoimmune diseases with the global prevalence estimated at 1%.^1^ Dietary gluten found in products containing wheat, barley, or rye, triggers an immune response in people with CD that damages the lining of the small intestines causing villous atrophy.^2^ As a result, people with CD can experience a wide range of symptoms from gastrointestinal symptoms to fatigue and weight loss which can vary greatly in severity. Long-term, this damage can lead to malabsorption, anaemia, osteoporosis, and in rare cases, cancer.^3^^,^^4^

Because symptoms are varied and non-specific, recognising CD is challenging and the majority of individuals with CD are not diagnosed. In the UK, it has been estimated that only one in three people with CD are diagnosed and that it takes 12 years on average to get the correct diagnosis.^5^^,^^6^ Currently, the only treatment is following a life-long gluten-free diet, which is effective in reducing symptoms and the risk of long-term complications.^7^^,^^8^ It is important that people with CD start this diet as soon as they are diagnosed to reverse the accumulated damage in the gut.^9^

Active case finding can help tackle underdiagnosis by offering CD tests to people at higher risk of CD, which has been shown to be a promising strategy.^10^ In a recent systematic review and meta-analysis, we identified a list of conditions that are predictive of having CD and should prompt testing.^11^ Our meta-analyses also suggested that symptoms on their own were not helpful in identifying patients with CD, as each symptom only increased the risk of CD by a small amount. It is unclear if, when used in combination, symptoms and risk conditions can be more helpful in identifying CD.

Here, we describe the development, internal and external validation of diagnostic prediction models for women, men, and children in routinely collected primary care datasets to estimate the probability of having CD. The aim of each prediction model is to help clinicians in primary care decide whether a patient should be offered a serological test for CD based on their pre-existing conditions and current or recent symptoms. To demonstrate the potential clinical usefulness of each model, we present the positive predictive values and percentage of people with CD missed at different thresholds.

## Methods

An analysis protocol was developed and published online (https://osf.io/q5gyc/). We followed methodological recommendations by Steyerberg (2019)^12^ and the Transparent Reporting of a multivariable prediction model for Individual Prognosis Or Diagnosis (TRIPOD) reporting guidelines.^13^

### Source of data

Model development was performed in Clinical Practice Research Datalink (CPRD) GOLD (data from Sep 9, 1987 to Apr 4, 2021);^14^ external validation was performed in CPRD Aurum (data from 738 practices, between Jan 1, 1995 and Jan 15, 2021).^15^ CPRD Gold contains anonymised patient electronic health records collected from UK GP practices using the Vision® software system, with currently over 20 million ‘acceptable’ patients (with research quality data based on CPRD metrics) of which 9 million are eligible for linkage with hospital records and national statistics.^14^^,^^16^ The included patients are broadly representative of the UK general population regarding age, sex, and ethnicity. CPRD Aurum contains electronic health records from GP practices in England using the EMIS® software system. CPRD Aurum is a larger dataset than CPRD Gold and contains over 40 million research acceptable patients of which 37 million are eligible for linkages with hospital records and national statistics.^17^ The included patients are broadly representative of the UK general population regarding age, sex, deprivation, and geographical spread. These datasets were linked to Hospital Episode Statistics (HES, data from 674 practices, 1997-2019) and English indices of deprivation (based on patient postcode, calculated in 2015).

### Participants

The GOLD extract included permanently registered ‘acceptable’ patients with up-to-standard (UTS) follow-up time.^16^ Included GP practices had to be UTS for at least 12 months prior to a patient's CD diagnosis. The follow-up period was defined as the time between the study start and end date, where the study start was the latest of the start of linked data coverage, the date of patient registration with the practice and the UTS date of that practice; the study end was the earliest of the last date for linked data, the date of patient transfer-out from practice, the date of patient's death or the last date of data collection from that practice. The same definitions were applied to the Aurum extract although Aurum does not report UTS, so this could not be taken into account when defining study start and end dates. Patients with records in both datasets were removed from the Aurum dataset based on their unique identifier. This study has been approved by the Independent Scientific Advisory Committee for MHRA database research (ISAC) (reference number 20_116A2). The ISAC protocol has been made available to the journal reviewers.

### Study design

We used a nested case control design. Cases and controls were matched using a 1:4 ratio on age group (age<18 and ≥18), GP practice, and availability of linkages. Controls inherited a pseudo-diagnosis date of their matched case and follow-up time was limited to match the case's follow-up time.

### Outcome

We developed separate models for men, women, and children. The models were developed to predict CD. CD was defined as the presence of one or more clinical codes related to CD that were developed in collaboration with clinicians (Supplementary Table S1). The first record of CD was taken as the date of diagnosis. Controls were individuals without these CD codes, and in additions we excluded patients with a record of gluten-free prescriptions, dermatitis herpetiformis, or gluten sensitivity diagnosis to reduce the risk of including undiagnosed CD patients as controls (Supplementary Table S1).^18^, ^19^, ^20^

### Predictors

#### Identifying candidate predictors

Predictors identified in our systematic review,^11^ CD guidelines,^21^, ^22^, ^23^ and predictors suggested by our clinical experts were considered for inclusion in the prediction models. Supplementary Table S2 presents the list of candidate predictors, their definitions, and how they were identified. International Classification of Primary Care 2 (ICPC-2) definitions were used where available. Dermatitis herpetiformis could not be included as an indicator because it was an exclusion criterion for the control cohort. Sex was considered as an indicator in the children's model and age was considered in all models.

#### Code list development

Existing code lists from publications were used if available, otherwise the CPRD code browser was used and codes were checked by at least two clinicians. The code lists developed for GOLD were mapped to medical codes used in Aurum using the CPRD code browser. The mapped lists were checked by hand before use.

### Sample size

To calculate the minimum total sample size and number of events, we used the R package “pmsamplesize()”^24^ which is based on the methods described by Riley et al.^25^ The input parameters were type (binary outcome, type=“b”), estimation of the R squared (rsquared = 0·1), number of parameters (candidate predictors, parameters = 40), shrinkage (shrinkage = 0·9), prevalence of outcome in our dataset (prevalence = 0·2), and seed (seed = 123). Because there are no previous models that are similar that could inform the R squared, we used a conservative value of 0·1, accepting a small absolute difference of 0·05 in the model's apparent and adjusted Nagelkerke's R-squared value. We used the value for shrinkage recommended by Riley and colleagues. This resulted in a minimum sample size required for new model development of 3397, with 680 events. Total sample sizes were at least four times larger in all three cohorts than this minimum.

### Missing data

It was not possible to determine whether a predictor was ‘missing’, because if medical codes were absent in a patient record, we assumed that the patient did not have the predictor in case of disease diagnoses or that the predictor was not considered sufficiently important to have been recorded by the GP in case of symptoms. Missingness was investigated for sex, ethnicity, and age; however, there were no missing data in these variables.

### Statistical analysis

We performed descriptive analyses of all variables and tested the statistical difference between cases and controls using the Welch Two Sample t-test for normally distributed continuous variables, Wilcoxon rank sum test for non-normally distributed continuous variables, and Pearson's Chi-squared test with Yates' continuity correction for categorical variables. The distribution of each variable was judged by visual inspection.

#### Model selection

We used elastic-net logistic regression models which perform both shrinkage and variable selection.^12^ It does this by including a regularization penalty (lambda) and a mixing parameter (alpha) where 0 results in ridge and 1 in lasso regression. Optimal alpha and lambda values were determined by testing 100 different lambda values at 18 different alpha values (increasing from 0·1 to 0·9). For each combination of alpha and lambda, twenty 5-fold cross-validations were performed. We selected the alpha-lambda combination that produced the model with the highest *c-*statistic (AUROC) which were fitted on 200 bootstrap samples. Predictors were selected based on the frequency of non-zero coefficients and the size and direction of the median value of each coefficient (i.e. predictors were dropped if they showed an inverse relationship with CD).

#### Model estimation

After estimating the optimal alpha and lambda, we re-fitted the elastic-net logistic regression model using the selected set of included predictors to determine the final coefficient estimates. No interaction terms were included. To estimate the intercept, we adjusted for sampling frequency for controls to recreate a population with the CD prevalence of the general population.^26^^,^^27^

#### Model performance

We estimated the model performance on the development dataset using measures of both discrimination and calibration.^28^ Discrimination is the ability of the model to distinguish between those with and without CD and was assessed using the *c*-statistic. Calibration is the agreement between predictions and observed outcomes. Calibration was assessed graphically using the calibration plot. We also assessed amount of variability explained by model variables with the Nagelkerke R-squared score and the overall model fit with the Brier score.^12^ We performed internal validation of the model using bootstrapping methods.^28^

#### Sensitivity analyses

We performed a sensitivity analysis restricting to patients diagnosed after 1997, when more accurate serological tests were introduced. Model development as described above was repeated on this dataset. We performed a second sensitivity analysis on datasets linked to HES and IMD2019 data to include ethnicity and deprivation in the models. We repeated the model development as described above on the subset of patients who were successfully linked to HES and IMD2019 data. We used the c-statistic to determine whether model performance was improved in each sensitivity analysis.

#### Clinical usefulness

We calculated the sensitivity and specificity of the prediction models for different thresholds of predicted CD risk. The thresholds were chosen based on the positive predictive values (PPV) of the models. The risk of CD amongst the general population is 1%, so we specified model thresholds that corresponded to PPVs of 1·5%, 2%, 5%, 10%, and 20%.

#### External validation

Predictions were made for the patients in Aurum using the intercepts and coefficients from the models developed in GOLD. Model performance statistics were calculated as described above. It was not possible to identify first-degree relatives with CD in Aurum. To account for this, we present all model performance measures as a range, across individuals with and individuals without a first-degree relative with CD.

#### Patient and Public Involvement statement

The study was designed with valuable input from two patient co-applicants who are 'experts by experience' being affected day to day by CD. As co-applicants for the project they contributed to provide input during the project proposal stage, attending project meetings to provide context from a patient viewpoint and providing feedback on research materials to ensure relevance to patient interests. They also reviewed and commented on the list of candidate predictors.

### Role of the funding source

The study sponsor was not involved in the study design; in the collection, analysis, and interpretation of data; in the writing of the report; nor in the decision to submit the paper for publication. All authors had access to the aggregated data in the study, and accept responsibility to submit for publication.

## Results

### Study participants

**Figure 1:**
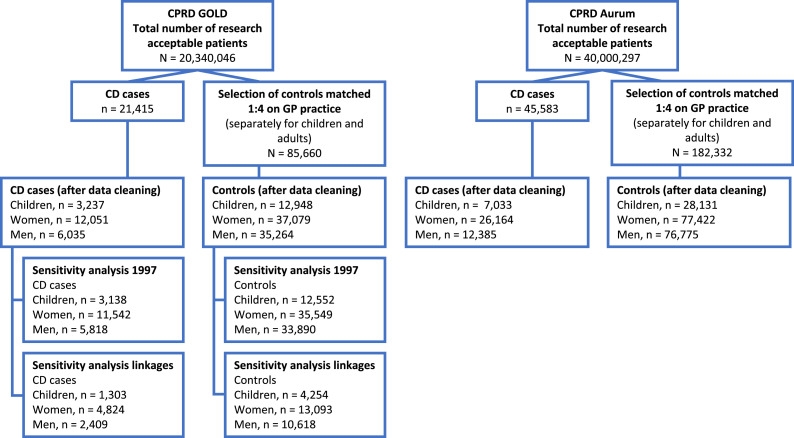
Patient flow diagram development (CPRD GOLD) and external validation dataset (CPRD Aurum). CPRD: Clinical Practice Research Datalink; CD: coeliac disease; GP: general practitioner.

Final datasets for model development contained 3,237 children, 12,051 women, and 6,035 men with CD and 12,948 children, 37,079 women, and 35,264 men as controls (Figure 1a). Datasets for external validation contained 7,033 children, 26,164 women, and 12,385 men with CD and 28,131 children, 77,422 women, and 76,775 men as controls (Figure 1b). Cases and controls had an average follow up time of 7 years prior to CD diagnosis (median 7, IQR 3-11 years, range 1-31 years).

In both child datasets, almost two thirds of those with CD were girls. Children and women with CD in Aurum were younger than controls, whereas men with CD were older than controls in both datasets. However, differences were small (Supplementary Tables S3-S5). Data on ethnicity and deprivation was available for a third of patients in GOLD and a fifth in Aurum. Amongst patients with known ethnicity, 90–95% were white and CD patients were more likely to be white than controls. People with CD in GOLD lived in more deprived areas (IMD quintile 1 and 2) than controls, which was not the case in Aurum. In GOLD, all predictors were more common in cases than in controls. In CPRD Aurum, arthritis and delayed puberty were not more common in children with CD (Supplementary Tables S3-S5). There were small differences in prevalences of predictors with most predictors being more prevalent in GOLD than in Aurum.

### Diagnostic indicator selection

The following candidate predictors could not be considered in the model because there were no observations with the respective codes: hyposplenism or functional asplenia, raised liver enzymes, multiple sclerosis, pancreatitis, pulmonary haemosiderosis, subfertility and recurrent pregnancy loss in children; delayed puberty and pulmonary haemosiderosis in women; amenorrhoea and turner syndrome in men. There were no observations of Williams-Beuren syndrome or dental enamel defects in any of the samples.

The following predictors showed an inverse relationship with CD and were dropped out of the model: amenorrhoea, arthritis, irritability, mood disorders, multiple sclerosis, subfertility, and type 2 diabetes for women, and type 2 diabetes for men. ADHD, headaches, migraines, hyposplenism or functional asplenia, IgA nephropathy, irritability, pancreatitis, type 2 diabetes, and multiple sclerosis were not selected as important predictors in any of the models (See Supplementary Table S6 for the proportion of non-zero coefficients per predictor and their median values across all bootstrap samples).

### Model specification

The optimal alpha and lambda values selected for the model for children were 0.004 and 0.75, for women 0.008 and 0.15, and for men 0.013 and 0.1.

For children, having type 1 diabetes, Turner syndrome, IgA deficiency, or a first-degree relative with CD were estimated to be the strongest predictors (i.e. had the highest estimated coefficients). For women and men the strongest predictors were having a first-degree relative with CD or anaemia. All three models included first-degree relatives with CD, anaemia, type 1 diabetes, iron, vitamin B12 or folate deficiency, thyroid disorders, weight loss, Down syndrome, gastrointestinal symptoms, fatigue, irritable bowel syndrome, and age. Epilepsy, cardiovascular disease, chronic liver disease, mouth ulcers, and osteoporosis were estimated to be important predictors for adults but not for children, whereas arthritis, failure to thrive, mood disorders, and delayed puberty were estimated to be predictive of CD in children but not in adults. Fractures, inflammatory bowel disorder, systemic lupus erythematosus, and neuropathy or ataxia were only selected predictors for women. See Supplementary Table S7-S9 for the intercepts, coefficients with and without shrinkage, and the adjusted and unadjusted ORs for each predictor.

### Model performance

**Figure 2:**
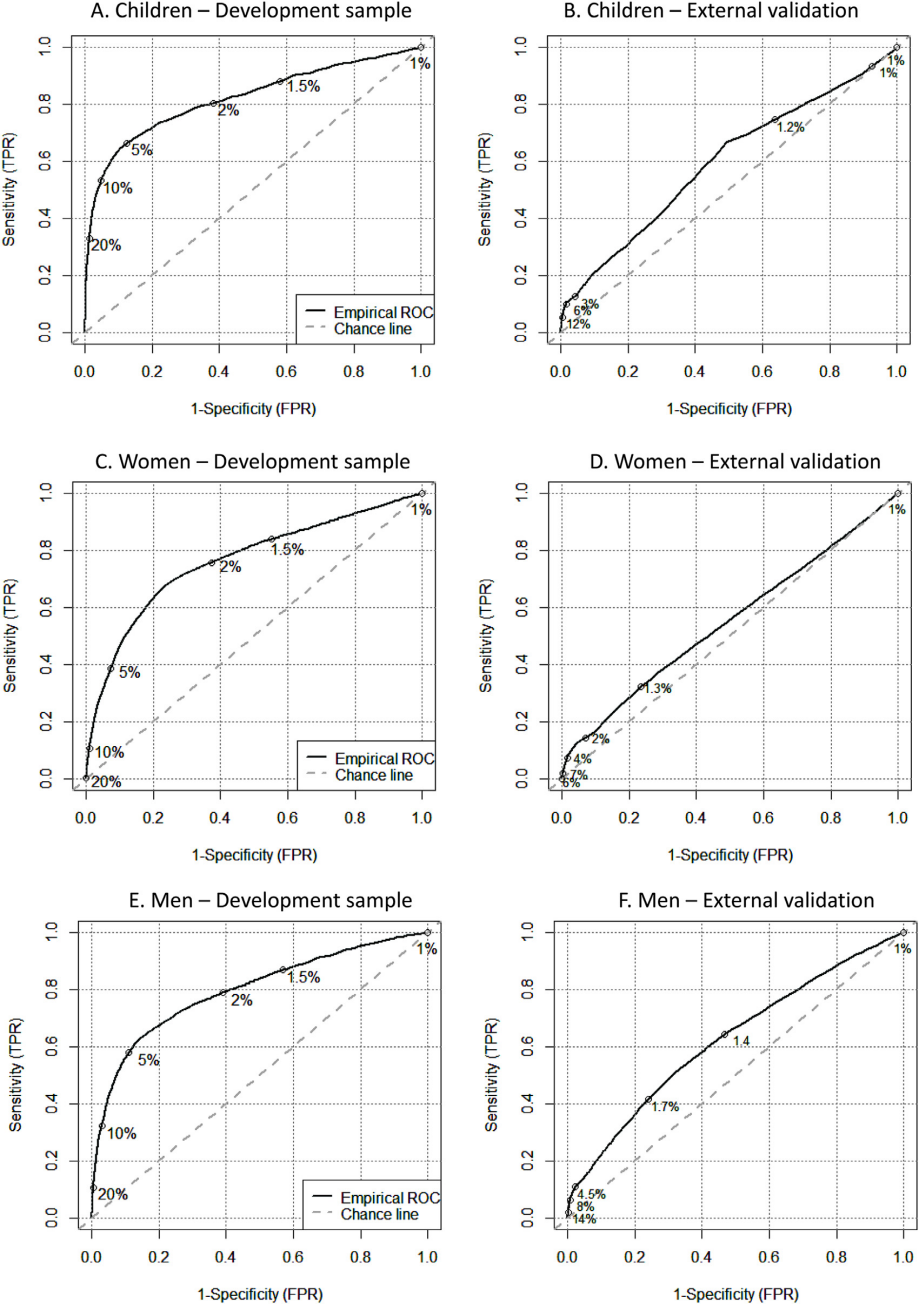
ROC curves model development. Thresholds are shown that result in a 1%, 1·5%, 2%, 5%, 10%, and 20% positive predictive value (PPV) for Children (A and B), for Women (C and D), and Men (E and F) in the development sample (CPRD GOLD) and the external data sample (CPRD Aurum), respectively. The same thresholds are applied on the external data. The black line represents the empirical ROC; the grey dashed line represents the chance line. FPR: false positive rate; TPR: true positive rate; ROC: receiver operating characteristic.

The development model in children shows the best overall model fit and ability to discriminate between those with and without CD compared to the models for men and women (Figure 2). Calibration curves are shown in Supplementary Figure S1. At higher risks, the model performs better. The estimated model performance shows to be stable, as the internal model performance in 200 bootstrap samples was similar with narrow confidence intervals (Table 1, Supplementary Table S10).

**Table 1:** Model performance.

	Apparent model performance	Internally validated model performance	Externally validated model performance
Data	Original data set (CPRD GOLD)	200x bootstrap samples of original data, median (IQR)	Independent data set (AURUM)
**Children**			
**R-squared**	0·407	0·408 (0·401; 0·413)	0·065
**Brier score**	0·167	0·167 (0·165; 0·169)	0·190 / 0·156 Without / with FDR
***C*-statistic**	0·821	0·821 (0·818; 0·824)	0·600
**Women**			
**R-squared**	0·237	0·248 (0·242; 0·254)	0·032
**Brier score**	0·227	0·225 (0·223; 0·227)	0·245 / 0·217 Without / with FDR
***C*-statistic**	0·756	0·764 (0·761; 0·767)	0·551
**Men**			
**R-squared**	0·286	0·284 (0·278; 0·291)	0·056
**Brier score**	0·122	0·124 (0·122; 0·126)	0·134 / 0·118 Without / with FDR
***C*-statistic**	0·798	0·796 (0·793;0·801)	0·619

CPRD: Clinical Practice Research Datalink; IQR: interquartile range; FDR: first-degree relative with coeliac disease.

### Sensitivity analyses

#### Sensitivity analysis on CD patients diagnosed after 1997

The vast majority of patients in the GOLD dataset were diagnosed after 1997, so limiting the analysis to these patients did not make a big impact on sample size. For this sensitivity analysis, 495 (3%) children, 2039 (4%) women, and 1591 (4%) men were removed from the respective datasets. Although there were minor changes in variable selection and model performance measures, the new models did not perform substantially better or worse than the original models.

#### Sensitivity analysis including ethnicity and deprivation as predictions

The linked dataset for children consisted of 4,254 controls and 1,303 CD patients, for women of 13,093 controls and 4,824 CD patients, and for men 10,618 controls and 2,409 CD patients. CD prevalence was higher in the linked datasets at 23.4%, 26.9%, and 18.5% for children, women, and men, respectively, compared to 20%, 24.5%, and 14.6% in the original datasets. Although ethnicity and IMD2015 quintiles were significantly associated with CD in all three samples, the updated model did not perform substantially better (Supplementary Table S12).

### External validation

The models performed less well in the validation dataset (Table 1). The amount of variability explained by the model dropped to below 7% in all models. The *c*-statistics were above 0·5, suggesting that the models discriminated better than chance. Calibration intercepts were further away from 0 and calibration slopes further away from 1 compared to the apparent model performance, indicating worse calibration.

### Clinical usefulness

The probability of CD from the prediction models of the prediction models can also be considered as the pre-test probability for serological testing, the next step in the diagnostic process. Currently in the UK, only one in three CD patients are believed to be diagnosed, so a prediction model that picks up more than one in three (i.e. sensitivity > 33%) might already improve case finding. The results suggest that this can be achieved at a pre-test probability of >20% for children, >5% in women, and >10% in men (Table 2). Table 3 shows examples of combinations of predictors in patients that reach these model thresholds.

**Table 2:** Clinical usefulness in development data.

Population	PPV	Threshold	TP	FP	FN	TN	Sens	Spec	NPV	% CD patients missed
**Children**	1%	0	100	9900	0	0	100·0%	0·0%	NA	0
	1·5%	0·0038	88	5776	12	4124	88·2%	41·7%	99·7%	11·8
	2%	0·0042	81	3865	19	6035	80·7%	61·0%	99·7%	19·3
	5%	0·0077	67	1271	33	8629	66·7%	87·2%	99·6%	33·3
	10%	0·0170	53	478	47	9422	53·3%	95·2%	99·5%	46·7
	20%*	0·0800	33	129	67	9771	33·1%	98·7%	99·3%	66·9

**Women**	1%	0	100	9900	0	0	100·0%	0·0%	NA	0
	1·5%	0·0053	84	5468	16	4432	84·1%	44·8%	99·6%	15·9
	2%	0·0062	76	3687	24	6213	75·8%	62·8%	99·6%	24·2
	5%*	0·0233	39	731	61	9169	38·7%	92·6%	99·3%	61·3
	10%	0·1070	11	96	89	9804	10·7%	99·0%	99·1%	89·3
	20%	0·7550	0	1	100	9899	0·2%	100·0%	99·0%	99·8

**Men**	1%	0	100	9900	0	0	100·0%	0·0%	NA	0
	1·5%	0·007	87	5634	13	4266	87·0%	43·1%	99·7%	13
	2%	0·008	79	3858	21	6042	79·0%	61·0%	99·7%	21
	5%	0·0185	58	1095	42	8805	57·9%	88·9%	99·5%	42·1
	10%*	0·0610	32	290	68	9610	32·2%	97·1%	99·3%	67·8
	20%	0·2820	11	43	89	9857	10·7%	99·6%	99·1%	89·3

In a population of 10,000 people.

⁎ PPVs that perform as good as current case finding in the UK, where only 1 in 3 people with CD are believed to be diagnosed. PPV: positive predictive value; TP: true positive; FP: false positive; FN: false negative; TN: true negative; Sens: sensitivity; Spec: specificity; NPV: negative predictive value; CD: coeliac disease.

**Table 3:** Examples of the combination of predictors in patients at several model thresholds.

Risk	Children	Women	Men
>1.5%	▪ All female children	▪ CVD ▪ Neuropathy or ataxia ▪ Fatigue* ▪ GI symptoms*	▪ Fatigue*

>2%	▪ Mood disorders ▪ GI symptoms* ▪ Fatigue*	▪ GI symptoms* and psoriasis ▪ CVD, GI symptoms* ▪ Chronic liver disease ▪ IBS ▪ Thyroid disease	▪ CVD ▪ IBS ▪ GI symptoms* ▪ Mouth ulcers* ▪ Epilepsy

>5%	▪ Fatigue within last year ▪ IBS ▪ Arthritis ▪ Failure to thrive	▪ Fatigue*, GI symptoms* and once last year, and IBS ▪ Anaemia ▪ Fatigue* and thyroid disorder ▪ FDR with CD	▪ GI symptoms*, and chronic liver disease or Epilepsy ▪ Down syndrome ▪ Weight loss

>10%	▪ GI symptoms* and once last year ▪ Failure to thrive and GI symptoms* ▪ Iron/folate/B12 deficiency ▪ Thyroid disorders ▪ Down syndrome ▪ Anaemia	▪ Anaemia, GI symptoms*, iron/folate/B12 deficiency ▪ GI symptoms* and 4 times last year, IBS ▪ Chronic liver disease, fatigue* and once last year, GI symptoms* and three times last year ▪ GI symptoms*, IBS, and osteoporosis	▪ GI symptoms* and twice last year ▪ T1D, fatigue*, GI symptoms* ▪ Fatigue, FDR ▪ GI symptoms*, osteoporosis ▪ Anaemia

>20%	▪ FDR with CD ▪ IgA deficiency ▪ Turner syndrome ▪ Type 1 diabetes	▪ Anaemia, fatigue*, GI symptoms* and four times last year, iron/B12/folate deficiency, thyroid disorder ▪ Anaemia, fatigue* and three times last year, GI symptoms* and twice last year, IBD, osteoporosis, and thyroid disorder ▪ Anaemia, CVD, GI symptoms* and 4 times last year, iron/B12/folate deficiency, weight loss	▪ Fatigue*, GI symptoms*, iron/B12/folate deficiency ▪ Fatigue* and once last year, GI symptoms*, thyroid disorders ▪ GI symptoms* and 4 times last year, IBS ▪ CVD, GI symptoms* and once last year, mouth ulcers* and twice last year

⁎ Symptoms that occurred within the last 10 years. CVD: cardiovascular disease; FDR: first-degree relative; GI: gastrointestinal; IBD: inflammatory bowel disease; IBS: irritable bowel syndrome; T1D: type 1 diabetes.

When applying the prediction model in CPRD Aurum, at the 20% threshold for children 95% of CD patients are missed, at the 5% threshold for women 86% CD patients are missed, and 10% for men 94% of CD patients are missed. However, lower thresholds still appear to be able to pick up more than the one in three CD patients (Figure 2, Supplementary Table S11).

## Discussion

We developed three prediction models to estimate the risk of having CD based on symptoms and risk factors that are available to a GP during a consultation. The final model for children included 24 predictors of which having type 1 diabetes, Turner syndrome, IgA deficiency, or a first-degree relative with CD were estimated to be the strongest predictors of CD. The models for women and men included 24, and 21 predictors, respectively, and the strongest predictors were having a first-degree relative with CD or anaemia. The models demonstrated good discrimination between patients with and without CD, but model performance was reduced in external validation. However, the external dataset did not report first-degree relatives, one of the most important predictors in each model, potentially leading to an underestimation of model performance in this dataset. All three models were poorly calibrated, tending to overestimate the risk of having CD in both the development and validation data. Investigating clinical usefulness of the models showed that a low threshold should be used for testing to improve case finding. This means that the presence of any single predictor is sufficient to warrant testing, suggesting that combining predictors into a prediction model is of limited value. However, we did identify several predictors that are not yet mentioned by CD guidelines to prompt testing. Combining the predictors into one model also showed their individual effect after taking other predictors into account.

This study has several strengths. We used robust definitions for predictors by using ICPC-2 definitions where available, which is the most widely used international classification for systematically capturing and ordering clinical information in primary care and is formally recognised by the World Health Organization's Family of International Classifications (WHO-FIC) as a classification system for primary care.^29^ Candidate predictors were prespecified based on an extensive literature review,^11^ instead of performing statistical variable selection only which risks instability of the selection, biased estimation of coefficients (testimation bias), misspecification of variability, and exaggeration of p-values.^12^ To avoid the effect of potential publication bias, we also included predictors suggested by our clinical experts and predictors listed in CD guidelines. As a second step, we used the elastic net method, which is a modern approach to variable selection using shrinkage, which optimises the variance (precision) and bias (accuracy) trade-off, to improve prediction in future data. Finally, the models were developed and externally validated in a large primary care dataset which makes the models more applicable and generalisable. The models are intended to be used in the primary care setting and GPs have access to the information needed for the models during consultation.

A major limitation of using routine CPRD data, however, is that CD is underdiagnosed and therefore underreported in CPRD. Diagnosed people with CD may have different characteristics than undiagnosed patients and different predictors may be important to detect currently undiagnosed CD patients. We were therefore more likely to confirm predictors that are already in the guidelines because those predictors currently prompt testing. In addition, some predictors were too rare to include in our model, such as Williams-Beuren syndrome, or were rarely recorded by GPs, such as dental enamel defects. This is a limitation of our model because both predictors are important according to several CD guidelines.^21^, ^22^, ^23^ We also relied on accurate recording of predictors by GPs and reporting by patients – non-specific symptoms such as gastrointestinal symptoms and fatigue are likely to be under-reported, so their true predictive ability cannot be estimated using primary care data. However, the models assess the predictive ability of these predictors as currently recorded by GPs in UK primary care, which is the information the GP has access to when making decisions about whether to test. A limitation of the study design for the prediction model is that we used a nested case control design. A cohort design is recommended for prediction modelling. We used recommended methods to estimate calibration statistics by artificially inflating the control group to recreate a CD prevalence similar to the general population. This method may have inflated any bias present in the original control group and might explain the poor calibration shown in our models. However, we believe this risk was low because our control group had a large sample size (>80,000 patients; large enough to reflect variation in all predictors) and controls had been randomly selected from a sample which is largely representative of the UK.

To our knowledge, this is the first prediction model using symptoms and chronic conditions to predict CD. However, genetic risk models using HLA and non-HLA variants as predictors of CD have been published.^30^^,^^31^ One model included both non-HLA genes and HLA risk genes and showed better classification then HLA risk genes alone.^30^ The model performance was improved by including more non-HLA genes resulting in a *c*-statistic of 0·85 (compared to 0·82 for HLA genes only).^31^ Sharp et al. developed a genetic risk score which was validated in data from the UK biobank and testing in a cohort of children with suspected coeliac disease.^32^ The genetic risk score performed better than using HLA-DQ typing, with a *c*-statistic of 0.88 [95% CIs: 0.87-0.89] in the UK biobank dataset and 0.84 [95% CIs: 0.76-0.91] in the pilot clinical cohort. Similar levels of discrimination could be achieved with simplified models including less single nucleotide polymorphisms as predictors.^33^ These models can help with assessing risk in at-risk groups; however, the main limitation of these models is that these genetic tests are not (yet) readily available to GPs.

When applying the models at a low risk threshold, e.g. 1·5% probability of CD, any single predictor is enough to push CD risk over the threshold and prompt testing. A few new predictors were identified that have not yet been adopted by guidelines or only by some. We identified mood disorders as important predictors for CD in children, which are not mentioned in current European paediatric guidelines.^23^ In addition, our results confirm the importance of offering CD testing to children with arthritis, chronic liver disease, and delayed puberty, which are currently mentioned as risk factors in the ESPGHAN (2020)^23^ guidelines but not in NICE.^21^ For women, we identified fractures (in addition to osteoporosis or pathological fractures) and for both men and women, cardiovascular disease as important predictors, which are not mentioned by any current guidelines. In additional, we could confirm several risk factors that are mentioned by the ESsCD (2019)^22^ guidelines but not by NICE: chronic liver disease, epilepsy, and psoriasis for both men and women and IgA deficiency and inflammatory bowel disease for women.

Future research should evaluate whether these models are cost-effective in improving case-finding to tackle underdiagnosis of CD. There is also a need for large prospective cohort studies where all participants receive accurate tests for CD to reduce bias in estimates of the diagnostic ability of predictors. Accurate testing strategies that don't rely on invasive tests such as a duodenal biopsy would make this more feasible. It is important that diagnostic prediction models use data in which all patients have been tested for CD to reduce bias as a result of underdiagnosis. This is essential to identify predictors for CD for patients who are currently not diagnosed, because routinely collected datasets are biased as they depend on current testing practices and are more likely to pick up predictors that are already used to prompts testing.

To help clinicians use prediction models in practice, these models can be fully integrated into GP software systems, so that they can flag up an increased risk of CD during a consultation. However, our analysis suggests that any single predictor is enough to increase CD risk to warrant testing. Offering a serological test to patients with any of these predictors has the potential to increase case finding for CD.

## Declaration of interests

All authors report funding from the National Institute for Health Research (NIHR) Health Technology Assessment Programme grant (NIHR129020). This publication presents independent research funded by the NIHR. The views expressed in this article are those of the author(s) and not necessarily those of the NIHR or the Department of Health and Social Care. Sue Mallett receives funding from the NIHR UCL/UCLH Biomedical Research Centre.

## Appendix Supplementary materials

Image, application 1

Image, application 2

Image, application 2
