# Supplementary material for: Development and external validation of a clinical prediction model to aid coeliac disease diagnosis in primary care: An observational study
Source: eClinicalMedicine. 2022 Apr 7;46:101376. doi: 10.1016/j.eclinm.2022.101376 (PMC9011008; doi:10.1016/j.eclinm.2022.101376)
Supplement: Supplementary file 1 [file mmc1.pdf]

Development and external validation of a clinical prediction model to aid coeliac disease diagnosis in primary care Supplementary materials

Authors
Martha M C Elwenspoek,[1,2] Rachel O'Donnell,[1,2] Joni Jackson,[1,2] Hazel Everitt,[4] Peter Gillett,[5] Alastair D Hay,[2] Hayley E Jones,[2] Gerry Robins,[7] Jessica C Watson,[2] Susan Mallett,[6] Penny Whiting.[2]

Affiliations
1. The National Institute for Health Research Applied Research Collaboration West (NIHR ARC West), University Hospitals Bristol NHS Foundation Trust, Bristol, BS1 2NT, UK
2. Population Health Sciences, Bristol Medical School, University of Bristol, Bristol, BS8 2PS, UK
3. Primary Care Research Centre, University of Southampton, Southampton SO16 5ST, UK
4. Paediatric Gastroenterology, Hepatology and Nutrition Department, Royal Hospital for Sick Children, Edinburgh EH9 1LF, Scotland, UK
5. Centre for Medical Imaging, University College London, 2nd Floor, Charles Bell House, 43-45 Foley Street, London, W1W 7TS, UK
6. Department of Gastroenterology, York Teaching Hospital NHS Foundation Trust, York, YO31 8HE, UK

Corresponding author
Martha M C Elwenspoek
9th Floor, Whitefriars, Lewins Mead,
Bristol, BS1 2NT
Email: Martha.Elwenspoek@bristol.ac.uk
Tel: +44/0 117 3427689

Email addresses co-authors
bb19384@bristol.ac.uk, joni.jackson@bristol.ac.uk, H.A.Everitt@soton.ac.uk, peter.gillett65@gmail.com, Alastair.Hay@bristol.ac.uk, Hayley.Jones@bristol.ac.uk, Gerry.Robins@york.nhs.uk, Jessica.Watson@bristol.ac.uk, sue.mallett@ucl.ac.uk, Penny.Whiting@bristol.ac.uk.

Contents

Methods 3

Participants 3

Table S1 Medical code lists for cases with coeliac disease and controls 3

Predictors 4

Table S2 Candidate diagnostic indicator definitions and sources 4

Transformations and categorisations of variables 7

Internal validation 7

Sensitivity analyses **Error! Bookmark not defined.**

Deviations from the protocol 7

Results 8

Model selection 8

Table S3 Predictor selection for children, women, and men 15

Model estimation 18

Table S4 Model coefficients and ORs with and without shrinkage applied - Children18

Table S5 Model coefficients and ORs with and without shrinkage applied - Women 19

Table S6 Model coefficients and ORs with and without shrinkage applied - Men 21

External validation 24

Table S7 Clinical usefulness in external validation data 26

Sensitivity analyses 26

Sensitivity analysis on CD patients diagnosed after 1997 **Error! Bookmark not defined.**

Sensitivity analysis including ethnicity and deprivation as predictions **Error! Bookmark not defined.**

Table S8 Model performance after including ethnicity and deprivation as predictions 26

References 27

Methods

Participants

Table S1 Medical code lists for cases with coeliac disease and controls

Term	Read code	MedCode
1. CD cases have at least one of the following codes		
Coeliac disease	J690·00	1515
Gluten enteropathy	J690·13	3509
Coeliac disease NOS	J690z00	44310
Acquired coeliac disease	J690100	63195
Sprue - nontropical	J690·14	16365
Congenital coeliac disease	J690000	62397
2. Controls have none of the following codes		
Coeliac disease	J690·00	1515
Gluten enteropathy	J690·13	3509
Coeliac disease NOS	J690z00	44310
Acquired coeliac disease	J690100	63195
Sprue - nontropical	J690·14	16365
Congenital coeliac disease	J690000	62397
Dermatitis herpetiformis	M140·00	3524
Gluten intolerance	J690·18	12217
Gluten-free diet	8B55·00	5662
Gluten-free diet	13B2·00	5664

Predictors

Table S2 Candidate diagnostic indicator definitions and sources

ICPC2: International Classification of Primary Care[1]

Predictors	Definition (ICPC2 definition where available)	Diagnostic indicator review	NICE 2015 guidelines	ESPGHAN 2020 guidelines	ESsCD 2019 guidelines
**Amenorrhea**	Primary and secondary amenorrhoea, i.e., the absence or cessation of menstruation			X	X
**Anaemia**	Any iron deficiency anaemia (B80), including anaemia due to blood loss.<br>Excludes: iron deficiency without anaemia T91	X		X	X
**Arthritis**	Includes: Rheumatoid/seropositive arthritis, allied condition: ankylosing spondylitis; allied condition: juvenile arthritis (L88)<br>Excludes: psoriatic arthropathy L99	X		X	
**Attention-deficit disorder/cognitive impairment**	Includes: hyperkinetic disorder, attention deficit disorder (ADD), hyperactivity (P81); cognitive impairment				X
**Cardiovascular disease**	Includes: Atherosclerosis/peripheral vascular disease, arterial embolism/thrombosis/stenosis; arteriosclerosis; atheroma; endarteritis; gangrene; intermittent claudication; limb ischaemia; Raynaud's syndrome; vasospasm (K92); Acute myocardial infarction (K75); Ischaemic heart disease with angina, angina of effort; angina pectoris; angina with spasm; ischaemic chest pain; unstable angina (K74); Ischaemic heart disease without angina, aneurysm of heart; arteriosclerotic/atherosclerotic heart disease; coronary artery disease; ischaemic cardiomyopathy; old myocardial infarction; silent myocardial ischaemia (K76); Stroke/cerebrovascular accident, apoplexy; cerebral embolism/infarction/thrombosis/occlusion/ stenosis/haemorrhage; cerebrovascular accident (CVA); subarachnoid haemorrhage (K90); Transient cerebral ischaemia, basilar insufficiency; drop attacks; transient global amnesia; transient ischaemic attack (TIA) (K89)	X			
**Chronic liver disease**	Includes: Liver disease NOS, alcohol hepatitis; cirrhosis; fatty liver; hepatitis NOS; liver failure; portal hypertension (D97); Viral hepatitis (D72)	X		X	X
**Delayed puberty**	Delayed puberty is when boys have no signs of testicular development by 14 years of age, girls have not started to develop breasts by 13 years of age, or they have developed breasts, but their periods have not started by 15 (NHS)			X	
**Dental enamel defects**	Enamel hypoplasia, dental enamel defects		X	X	X
**Down syndrome**	Down syndrome		X	X	X
**Epilepsy**	Includes: All types of epilepsy, focal seizures; generalized seizures; grand mal; petit mal; status epilepticus (N88), convulsion N07	X			X
**Failure to thrive**	Includes: failure to thrive, physiological delay growth (T10)<br>Excludes: delayed milestones P22; learning disorder P24; mental retardation P85; delayed puberty T99		X	X	X
**Fatigue**	Includes: Weakness/tiredness general, chronic fatigue syndrome; exhaustion; fatigue; lassitude; lethargy; post viral fatigue (A04)<br>Excludes: malaise/feeling ill A05; drowsiness A29; heat exhaustion A88; jetlag A88; Systemic lupus erythematosusep disturbance P06		X	X	X
**First-degree relatives of CD**	Parent, sibling, or child with coeliac disease	X	X	X	X
**Fractures**	Includes: radius/ulna fracture (L72), tibia/fibula fracture (L73), hand/foot bone fracture (L74), femur fracture (L75), other fractures (76)<br>Excludes: pathological fracture (osteoporosis) L95; pathological fracture NOS L99; non-union L99	X		X	

Predictors	Definition (ICPC2 definition where available)	Diagnostic indicator review	NICE 2015 guidelines	ESPGHAN 2020 guidelines	ESsCD 2019 guidelines
**Gastrointestinal symptoms**	Includes: abdominal colic; abdominal cramps/discomfort/pain NOS; infant colic (D01), heartburn, acidity, waterbrash (D03), epigastric pain (D02); dyspepsia/indigestion (D07); oesophagitis/reflux (D84), flatulence/gas/belching D08, bloating; eructation; gas pains; gaseous distension; passing wind; or abdominal distension (abdominal swelling without mass) D25, Constipation, faecal impaction (D12), Diarrhoea, frequent/loose bowel movements; watery stools (D11), Vomiting, emesis; hyperemesis; retching (D10); Nausea (D09).<br>Excludes: epigastric ache D02; other localized abdominal pain D06; biliary colic D98; renal colic U14; dysmenorrhoea X02, abdominal mass D24; ascites D29, ileus D99, melaena D15; change in faeces/bowel movements D18, haematemesis D14; vomiting in pregnancy W05, feelings of over-eating D02;alcohol induced nausea P16; loss of appetite T03; nausea in pregnancy W05.	X	X	X	X
**Hyposplenism or functional asplenia**	Hyposplenism (reduced splenic functioning) or functional asplenia (absence of normal spleen function), including splenectomy				X
**IgA deficiency**	IgA deficiency			X	X
**IgA nephropathy**	Also known as Berger's disease				X
**Inflammatory bowel disease**	Inflammatory bowel disease (IBD) is a term for two conditions (Crohn's disease and ulcerative colitis) that are characterized by chronic inflammation of the gastrointestinal (GI) tract.	X			X
**Iron, vitamin B12, or folate deficiency**	Includes: Anaemia vit B12/folate deficiency, macrocytic anaemia, pernicious anaemia (B81); vit B12 deficiency without anaemia T91, iron deficiency without anaemia		X		
**Irritability**	Includes: Feeling/behaving irritable/angry, agitation NOS; restlessness NOS (P04)<br>Excludes: overactive child P22; irritability in partner Z13			X	
**Irritable bowel syndrome**	Includes: Irritable bowel syndrome (D93), spastic colon<br>Excludes: gastrointestinal infection D70; gastroenteritis presumed infection D73; regional enteritis D94; allergic/dietetic/toxic gastroenteritis/colitis D99; vascular insufficiency of gut D99; psychogenic diarrhoea P75	X	X		X
**Migraine or headaches**	Includes: Headache, post-traumatic headache (N01); migraine N89; cluster headache N90; tension headache N95<br>Excludes: cervicogenic headache L83; face pain N03; atypical facial neuralgia N99; sinus pain R09; post-herpetic pain S70	X			X
**Mood disorders**	Includes: Depressive disorder, depressive neurosis/psychosis; mixed anxiety and depression; puerperal/postnatal depression; reactive depression (P76); affective psychosis, bipolar disorder; hypomania; mania; manic depression (P73)				X
**Multiple sclerosis**	Includes: Multiple sclerosis, disseminated sclerosis (N86)	X			
**Neuropathy or ataxia**	Includes: Peripheral neuritis/neuropathy, acute infective polyneuropathy; diabetic neuropathy (double code with T89, T90); Guillain-Barre syndrome; nerve lesion; neuropathy; phantom limb (N94); Neurological symptom/complaint other, ataxia; gait abnormality; limping; meningism (N29)		X	X	X
**Osteoporosis**	Includes: osteoporosis, pathological fracture due to osteoporosis (L95); Osteomalacia, osteopenia, decreased bone mineralization	X	X	X	X
**Pancreatitis**	Unexplained acute or chronic pancreatitis				X
**Psoriasis**	Psoriasis (S91)	X			X
**Pulmonary haemosiderosis**	Pulmonary haemosiderosis				X
**Raised liver enzymes**	Elevated liver enzymes (including Alanine transaminase (ALT), Aspartate transaminase (AST), Alkaline phosphatase (ALP), Gamma-glutamyl transpeptidase (GGT)	X	X	X	X

Predictors	Definition (ICPC2 definition where available)	Diagnostic indicator review	NICE 2015 guidelines	ESPGHAN 2020 guidelines	ESsCD 2019 guidelines
**Severe or persistent mouth ulcers**	Severe or persistent mouth ulcers including recurrent aphthous stomatitis		X	X	X
**Subfertility or recurrent miscarriage**	Includes: Abortion spontaneous, abortion complete/incomplete/missed/habitual, miscarriage (W82); Infertility/subfertility female, primary and secondary sterility (W15)	X	X		X
**Systemic lupus erythematosus**	Systemic lupus erythematosus	X			
**Thyroid disease**	Thyroiditis, autoimmune Thyroiditis, hypothyroidism, hyperthyroidism, Graves' disease, goiter, Hashimoto thyroiditis, Painless thyroiditis (silent thyroiditis), Subacute thyroiditis, Graves' disease, excluding Postpartum thyroiditis	X	X	X	X
**Turner syndrome**	Turner syndrome		X	X	X
**Type 1 Diabetes**	Type 1 Diabetes, includes juvenile diabetes or insulin-dependent diabetes (T89)	X	X	X	
**Type 2 Diabetes**	Type 2 Diabetes, includes diabetes NOS; late onset diabetes; type 2 diabetes (T90)	X			
**Weight loss**	Includes: Weight loss, cachexia (T08)	X	X	X	X
	Excludes: anorexia nervosa P86				
**Williams-Beuren syndrome**	Also known as Williams syndrome			X	

Transformations and categorisations of variables
Age was included in the model as a linear term (in years), because the risk of being diagnosed decreases linearly by age.2 Ethnicity was transformed to a binary variable. Deprivation quintiles were used as deprivation score.

All risk conditions were coded as binary variables, where 1 is having the disease at any timepoint prior to CD diagnosis. Predictors that can resolve and return (GI symptoms, weight loss, fatigue, abnormal liver function test results, mouth ulcers, irritability, iron, vitamin B12, or folate deficiency, fractures, and headaches or migraines) were coded as 1 if the event occurred within 10 years prior to CD diagnosis. Predictors that can vary substantially over time (GI symptoms, fatigue, irritability, mouth ulcers, fractures, and migraine or headaches) were also included as counts (incidence within 1, 2, or 10 years prior to CD diagnosis).

We defined people as having a first-degree relative with CD if their unique *famnum* (by combining *famnum* and GP practice IDs) was identical to that of a CD patient, and if both were either <25 years old and differed less than 15 years in age or differed more than 15 years in age.

Internal validation
We performed internal validation of the model using bootstrapping methods.[3] We fitted the final model using elastic net regression with the predefined optimal lambda and alpha values on 1000 bootstrap samples to estimate the median of each coefficient and calculate empirical confidence intervals around the coefficients. The elastic net regression uses shrinkage to adjust for overfitting and optimism. Using the 1000 model fits, we calculated the median and empirical confidence intervals for performance statistics (R-squared, Brier score, and c statistic). To estimate the median and empirical confidence intervals for the intercept and calibration statistics, we adjusted for sampling frequency by recreating a population with the CD prevalence of the general population.[4,5]

Deviations from the protocol
In the CPRD model development, we used logistic regression instead of conditional logistic regression because cases and controls were matched on very few characteristics, namely being a child or an adult and GP practice. Interaction terms were not considered as they are rarely important for clinical prediction models.[6] We planned to validate the CPRD model in children in the ALSPAC cohort. However, this was not possible because many of the selected predictors in the CPRD model were not recorded in ALSPAC and among the predictors that were available there was a lot of missingness.

Results

Study participants

Table S3 Participant characteristics of cohort of children

P values show the result of a Wilcoxon rank sum test for continuous and Pearson's Chi-squared test with Yates' continuity correction for categorical variables.

	Development dataset (CPRD GOLD)				External validation dataset (CPRD Aurum)			
CHILDREN	Control (N=12948)	Coeliac disease (N=3237)	P values	Overall (N=16185)	Control (N=28131)	Coeliac disease (N=7033)	P values	Overall (N=35164)
**Age (years)**								
Mean (SD)	9·04 (4·62)	8·89 (4·77)	0·127	9·01 (4·65)	8·96 (4·61)	8·66 (4·74)	0.103	8·90 (4·64)
Median [Min, Max]	9·00 [1·00, 17·0]	9·00 [1·00, 17·0]		9·00 [1·00, 17·0]	9·00 [1·00, 17·0]	8·00 [1·00, 17·0]		9·00 [1·00, 17·0]
**Sex**								
Male	6862 (53·0%)	1249 (38·6%)	<0·001	8111 (50·1%)	15024 (53·4%)	2702 (38·4%)	<0·001	17726 (50·4%)
Female	6086 (47·0%)	1988 (61·4%)		8074 (49·9%)	13107 (46·6%)	4331 (61·6%)		17438 (49·6%)
**Ethnicity**								
Non-white	438 (3·4%)	98 (3·0%)	0·004	536 (3·3%)	686 (2·4%)	156 (2·2%)	<0·001	842 (2·4%)
White	3816 (29·5%)	1205 (37·2%)		5021 (31·0%)	3478 (12·4%)	1103 (15·7%)		4581 (13·0%)
Missing	8694 (67·1%)	1934 (59·7%)		10628 (65·7%)	23967 (85·2%)	5774 (82·1%)		29741 (84·6%)
**Deprivation (IMD 2015 quintiles)**								
1	1104 (8·5%)	385 (11·9%)	0·059	1489 (9·2%)	1532 (5·4%)	391 (5·6%)	0·057	1923 (5·5%)
2	896 (6·9%)	270 (8·3%)		1166 (7·2%)	1154 (4·1%)	310 (4·4%)		1464 (4·2%)
3	871 (6·7%)	264 (8·2%)		1135 (7·0%)	1000 (3·6%)	280 (4·0%)		1280 (3·6%)
4	742 (5·7%)	219 (6·8%)		961 (5·9%)	836 (3·0%)	188 (2·7%)		1024 (2·9%)
5	641 (5·0%)	165 (5·1%)		806 (5·0%)	858 (3·1%)	185 (2·6%)		1043 (3·0%)
Missing	8694 (67·1%)	1934 (59·7%)		10628 (65·7%)	22751 (80·9%)	5679 (80·7%)		28430 (80·8%)
**Anaemia**								
Present	46 (0·4%)	188 (5·8%)	<0·001	234 (1·4%)	25 (0·1%)	84 (1·2%)	<0·001	109 (0·3%)
**Arthritis**								
Present	5 (0·0%)	7 (0·2%)	0·003	12 (0·1%)	5 (0·0%)	3 (0·0%)	0·426	8 (0·0%)
**Delayed puberty**								

	Development dataset (CPRD GOLD)				External validation dataset (CPRD Aurum)			
Present	1 (0·0%)	5 (0·2%)	<0·001	6 (0·0%)	2 (0·0%)	2 (0·0%)	0·382	4 (0·0%)
**Down syndrome**								
Present	6 (0·0%)	18 (0·6%)	<0·001	24 (0·1%)	2 (0·0%)	15 (0·2%)	<0·001	17 (0·0%)
**Failure to thrive**								
Present	52 (0·4%)	84 (2·6%)	<0·001	136 (0·8%)	21 (0·1%)	31 (0·4%)	<0·001	52 (0·1%)
**Fatigue**								
Present	210 (1·6%)	253 (7·8%)	<0·001	463 (2·9%)	165 (0·6%)	150 (2·1%)	<0·001	315 (0·9%)
**Fatigue (count, 1 year)**								
Once	53 (0·4%)	130 (4·0%)	<0·001	183 (1·1%)	34 (0·1%)	48 (0·7%)	<0·001	82 (0·2%)
Twice	5 (0·0%)	17 (0·5%)		22 (0·1%)	8 (0·0%)	14 (0·2%)		22 (0·1%)
Three times	0 (0%)	11 (0·3%)		11 (0·1%)	2 (0·0%)	8 (0·1%)		10 (0·0%)
**First-degree relative**								
Present	98 (0·8%)	496 (15·3%)	<0·001	594 (3·7%)	NR	NR		NR
**GI symptoms**								
Present	3684 (28·5%)	1924 (59·4%)	<0·001	5608 (34·6%)	1371 (4·9%)	794 (11·3%)	<0·001	2165 (6·2%)
**GI symptoms (count, 1 year)**								
Once	785 (6·1%)	588 (18·2%)	<0·001	1373 (8·5%)	293 (1·0%)	218 (3·1%)	<0·001	511 (1·5%)
Twice	192 (1·5%)	294 (9·1%)		486 (3·0%)	82 (0·3%)	126 (1·8%)		208 (0·6%)
Three times	55 (0·4%)	154 (4·8%)		209 (1·3%)	24 (0·1%)	60 (0·9%)		84 (0·2%)
Four times	52 (0·4%)	203 (6·3%)		255 (1·6%)	39 (0·1%)	98 (1·4%)		137 (0·4%)
**Irritable bowel syndrome**								
Present	20 (0·2%)	29 (0·9%)	<0·001	49 (0·3%)	6 (0·0%)	12 (0·2%)	<0·001	18 (0·1%)
**IgA deficiency**								
Present	0 (0%)	4 (0·1%)	<0·001	4 (0·0%)	1 (0·0%)	4 (0·1%)	0·005	5 (0·0%)
**Iron, vit B12, or folate deficiency**								
Present	8 (0·1%)	35 (1·1%)	<0·001	43 (0·3%)	8 (0·0%)	24 (0·3%)	<0·001	32 (0·1%)
**Mood disorders**								
Present	256 (2·0%)	143 (4·4%)	<0·001	399 (2·5%)	91 (0·3%)	59 (0·8%)	<0·001	150 (0·4%)

	Development dataset (CPRD GOLD)				External validation dataset (CPRD Aurum)			
**Type 1 diabetes**								
Present	16 (0·1%)	275 (8·5%)	<0·001	291 (1·8%)	11 (0·0%)	87 (1·2%)	<0·001	98 (0·3%)
**Thyroid disorders**								
Present	16 (0·1%)	61 (1·9%)	<0·001	77 (0·5%)	12 (0·0%)	21 (0·3%)	<0·001	33 (0·1%)
**Turner syndrome**								
Present	0 (0%)	8 (0·2%)	<0·001	8 (0·0%)	1 (0·0%)	5 (0·1%)	<0·001	6 (0·0%)
**Weight loss**								
Present	23 (0·2%)	93 (2·9%)	<0·001	116 (0·7%)	8 (0·0%)	22 (0·3%)	<0·001	30 (0·1%)

Table S4 Participant characteristics of cohort of women

P values show the result of a Welch Two Sample t-test for continuous and Pearson's Chi-squared test with Yates' continuity correction for categorical variables.

	Development dataset (CPRD GOLD)				External validation dataset (CPRD Aurum)			
**WOMEN**	**Control (N=37079)**	**Coeliac disease (N=12051)**	**P values**	**Overall (N=49130)**	**Control (N=77422)**	**Coeliac disease (N=26164)**	**P values**	**Overall (N=103586)**
**Age (years)**								
Mean (SD)	49·5 (17·0)	49·7 (17·2)	0·313	49·6 (17·0)	48·5 (17·0)	47·4 (17·6)	<0·001	48·2 (17·2)
Median [Min, Max]	49·0 [18·0, 111]	49·0 [18·0, 104]		49·0 [18·0, 111]	47·0 [18·0, 108]	46·0 [18·0, 99·0]		47·0 [18·0, 108]
**Ethnicity**								
Non-white	807 (2·2%)	212 (1·8%)	<0·001	1019 (2·1%)	1262 (1·6%)	399 (1·5%)	<0·001	1661 (1·6%)
White	12286 (33·1%)	4612 (38·3%)		16898 (34·4%)	11210 (14·5%)	4496 (17·2%)		15706 (15·2%)
Missing	23986 (64·7%)	7227 (60·0%)		31213 (63·5%)	64950 (83·9%)	21269 (81·3%)		86219 (83·2%)
**Deprivation (IMD 2015 quintiles)**								
1	3199 (8·6%)	1262 (10·5%)	0·174	4461 (9·1%)	4255 (5·5%)	1424 (5·4%)	0·276	5679 (5·5%)
2	2939 (7·9%)	1077 (8·9%)		4016 (8·2%)	3696 (4·8%)	1254 (4·8%)		4950 (4·8%)
3	2761 (7·4%)	1001 (8·3%)		3762 (7·7%)	2975 (3·8%)	991 (3·8%)		3966 (3·8%)
4	2292 (6·2%)	819 (6·8%)		3111 (6·3%)	2642 (3·4%)	966 (3·7%)		3608 (3·5%)
5	1902 (5·1%)	665 (5·5%)		2567 (5·2%)	2146 (2·8%)	702 (2·7%)		2848 (2·7%)

	Development dataset (CPRD GOLD)				External validation dataset (CPRD Aurum)			
Missing	23986 (64·7%)	7227 (60·0%)		31213 (63·5%)	61708 (79·7%)	20827 (79·6%)		82535 (79·7%)
**Anaemia**								
Present	1038 (2·8%)	1969 (16·3%)	<0·001	3007 (6·1%)	484 (0·6%)	968 (3·7%)	<0·001	1452 (1·4%)
**Cardiovascular disease**								
Present	1601 (4·3%)	883 (7·3%)	<0·001	2484 (5·1%)	602 (0·8%)	317 (1·2%)	<0·001	919 (0·9%)
**Chronic liver disease**								
Present	376 (1·0%)	253 (2·1%)	<0·001	629 (1·3%)	233 (0·3%)	180 (0·7%)	<0·001	413 (0·4%)
**Down syndrome**								
Present	5 (0·0%)	5 (0·0%)	0·132	10 (0·0%)	4 (0·0%)	4 (0·0%)	0·229	8 (0·0%)
**Epilepsy**								
Present	194 (0·5%)	116 (1·0%)	<0·001	310 (0·6%)	109 (0·1%)	46 (0·2%)	0·24	155 (0·1%)
**Fatigue**								
Present	4638 (12·5%)	3027 (25·1%)	<0·001	7665 (15·6%)	1755 (2·3%)	1165 (4·5%)	<0·001	2920 (2·8%)
**Fatigue (count, 1 year)**								
Once	945 (2·5%)	953 (7·9%)	<0·001	1898 (3·9%)	333 (0·4%)	341 (1·3%)	<0·001	674 (0·7%)
Twice	145 (0·4%)	174 (1·4%)		319 (0·6%)	86 (0·1%)	118 (0·5%)		204 (0·2%)
Three times	38 (0·1%)	73 (0·6%)		111 (0·2%)	56 (0·1%)	79 (0·3%)		135 (0·1%)
**First-degree relative**								
Present	108 (0·3%)	416 (3·5%)	<0·001	524 (1·1%)	NR	NR		NR
**Fractures (count, 1 year)**								
Once	320 (0·9%)	202 (1·7%)	<0·001	522 (1·1%)	139 (0·2%)	65 (0·2%)	<0·001	204 (0·2%)
Twice	169 (0·5%)	84 (0·7%)		253 (0·5%)	92 (0·1%)	60 (0·2%)		152 (0·1%)
**GI symptoms**								
Present	12364 (33·3%)	6994 (58·0%)	<0·001	19358 (39·4%)	4520 (5·8%)	2603 (9·9%)	<0·001	7123 (6·9%)
**GI symptoms (count, 1 year)**								
Once	2605 (7·0%)	2206 (18·3%)	<0·001	4811 (9·8%)	949 (1·2%)	774 (3·0%)	<0·001	1723 (1·7%)
Twice	773 (2·1%)	996 (8·3%)		1769 (3·6%)	310 (0·4%)	349 (1·3%)		659 (0·6%)
Three times	308 (0·8%)	507 (4·2%)		815 (1·7%)	126 (0·2%)	179 (0·7%)		305 (0·3%)

	Development dataset (CPRD GOLD)				External validation dataset (CPRD Aurum)			
Four times	265 (0·7%)	679 (5·6%)		944 (1·9%)	162 (0·2%)	302 (1·2%)		464 (0·4%)
**Inflammatory bowel disease**								
Present	160 (0·4%)	104 (0·9%)	<0·001	264 (0·5%)	82 (0·1%)	54 (0·2%)	<0·001	136 (0·1%)
**Irritable bowel syndrome**								
Present	1716 (4·6%)	1346 (11·2%)		3062 (6·2%)	757 (1·0%)	545 (2·1%)	<0·001	1302 (1·3%)
**IgA deficiency**								
Present	2 (0·0%)	6 (0·0%)	0·004	8 (0·0%)	1 (0·0%)	7 (0·0%)	<0·001	8 (0·0%)
**Iron, vit B12, or folate deficiency**								
Present	505 (1·4%)	938 (7·8%)	<0·001	1443 (2·9%)	235 (0·3%)	394 (1·5%)	<0·001	629 (0·6%)
**Mouth ulcers (count, 1 year)**								
Once	110 (0·3%)	121 (1·0%)	<0·001	231 (0·5%)	43 (0·1%)	32 (0·1%)	<0·001	75 (0·1%)
Twice	12 (0·0%)	38 (0·3%)		50 (0·1%)	11 (0·0%)	16 (0·1%)		27 (0·0%)
**Neuropathy or ataxia**								
Present	84 (0·2%)	55 (0·5%)	<0·001	139 (0·3%)	56 (0·1%)	36 (0·1%)	0·003	92 (0·1%)
**Osteoporosis**								
Present	915 (2·5%)	898 (7·5%)	<0·001	1813 (3·7%)	367 (0·5%)	305 (1·2%)	<0·001	672 (0·6%)
**Systemic lupus erythematosus**								
Present	42 (0·1%)	40 (0·3%)	<0·001	82 (0·2%)	23 (0·0%)	18 (0·1%)	<0·001	41 (0·0%)
**Type 1 diabetes**								
Present	99 (0·3%)	141 (1·2%)	<0·001	240 (0·5%)	223 (0·3%)	147 (0·6%)	<0·001	370 (0·4%)
**Thyroid disorders**								
Present	2042 (5·5%)	1442 (12·0%)	<0·001	3484 (7·1%)	815 (1·1%)	623 (2·4%)	<0·001	1438 (1·4%)
**Turner syndrome**								
Present	3 (0·0%)	5 (0·0%)	0·037	8 (0·0%)	0 (0%)	5 (0·0%)	<0·001	5 (0·0%)
**Weight loss**								
Present	500 (1·3%)	672 (5·6%)	<0·001	1172 (2·4%)	105 (0·1%)	145 (0·6%)	<0·001	250 (0·2%)

Table S5 Participant characteristics of cohort of men

P values show the result of a Welch Two Sample t-test for continuous and Pearson's Chi-squared test with Yates' continuity correction for categorical variables.

	Development dataset (CPRD GOLD)				External validation dataset (CPRD Aurum)			
**MEN**	**Control (N=35264)**	**Coeliac disease (N=6035)**	**P values**	**Overall (N=41299)**	**Control (N=76775)**	**Coeliac disease (N=12385)**	**P values**	**Overall (N=89160)**
**Age (years)**								
Mean (SD)	47·5 (16·4)	53·9 (16·3)	<0·001	48·4 (16·6)	46·6 (16·5)	52·4 (17·1)	<0·001	47·4 (16·7)
Median [Min, Max]	47·0 [18·0, 103]	55·0 [18·0, 94·0]		48·0 [18·0, 103]	46·0 [18·0, 107]	53·0 [18·0, 98·0]		47·0 [18·0, 107]
**Ethnicity**								
Non-white	535 (1·5%)	94 (1·6%)	0·022	629 (1·5%)	976 (1·3%)	155 (1·3%)	<0·001	1131 (1·3%)
White	10083 (28·6%)	2315 (38·4%)		12398 (30·0%)	8967 (11·7%)	2100 (17·0%)		11067 (12·4%)
Missing	24646 (69·9%)	3626 (60·1%)		28272 (68·5%)	66832 (87·0%)	10130 (81·8%)		76962 (86·3%)
**Deprivation (IMD 2015 quintiles)**								
1	2563 (7·3%)	624 (10·3%)	0·005	3187 (7·7%)	4205 (5·5%)	682 (5·5%)	0·55	4887 (5·5%)
2	2389 (6·8%)	597 (9·9%)		2986 (7·2%)	3745 (4·9%)	625 (5·0%)		4370 (4·9%)
3	2264 (6·4%)	450 (7·5%)		2714 (6·6%)	3038 (4·0%)	478 (3·9%)		3516 (3·9%)
4	1852 (5·3%)	399 (6·6%)		2251 (5·5%)	2745 (3·6%)	431 (3·5%)		3176 (3·6%)
5	1550 (4·4%)	339 (5·6%)		1889 (4·6%)	2139 (2·8%)	316 (2·6%)		2455 (2·8%)
Missing	24646 (69·9%)	3626 (60·1%)		28272 (68·5%)	60903 (79·3%)	9853 (79·6%)		70756 (79·4%)
**Anaemia**								
Present	208 (0·6%)	733 (12·1%)	<0·001	941 (2·3%)	104 (0·1%)	305 (2·5%)	<0·001	409 (0·5%)
**Cardiovascular disease**								
Present	2081 (5·9%)	860 (14·3%)	<0·001	2941 (7·1%)	822 (1·1%)	321 (2·6%)	<0·001	1143 (1·3%)
**Chronic liver disease**								
Present	421 (1·2%)	184 (3·0%)	<0·001	605 (1·5%)	311 (0·4%)	140 (1·1%)	<0·001	451 (0·5%)
**Down syndrome**								
Present	6 (0·0%)	6 (0·1%)	0·002	12 (0·0%)	1 (0·0%)	4 (0·0%)	<0·001	5 (0·0%)
**Epilepsy**								
Present	207 (0·6%)	69 (1·1%)	<0·001	276 (0·7%)	101 (0·1%)	45 (0·4%)	<0·001	146 (0·2%)

	Development dataset (CPRD GOLD)				External validation dataset (CPRD Aurum)			
**Fatigue**								
Present	1941 (5·5%)	881 (14·6%)	<0·001	2822 (6·8%)	710 (0·9%)	346 (2·8%)	<0·001	1056 (1·2%)
**Fatigue (count, 1 year)**								
Once	396 (1·1%)	288 (4·8%)	<0·001	684 (1·7%)	113 (0·1%)	87 (0·7%)	<0·001	200 (0·2%)
Twice	50 (0·1%)	63 (1·0%)		113 (0·3%)	28 (0·0%)	35 (0·3%)		63 (0·1%)
Three times	17 (0·0%)	22 (0·4%)		39 (0·1%)	15 (0·0%)	34 (0·3%)		49 (0·1%)
**First-degree relative**								
Present	103 (0·3%)	157 (2·6%)	<0·001	260 (0·6%)	NR	NR		NR
**GI symptoms**								
Present	7850 (22·3%)	3164 (52·4%)	<0·001	11014 (26·7%)	2974 (3·9%)	1154 (9·3%)	<0·001	4128 (4·6%)
**GI symptoms (count, 1 year)**								
Once	1482 (4·2%)	1030 (17·1%)	<0·001	2512 (6·1%)	535 (0·7%)	306 (2·5%)	<0·001	841 (0·9%)
Twice	421 (1·2%)	483 (8·0%)		904 (2·2%)	170 (0·2%)	172 (1·4%)		342 (0·4%)
Three times	134 (0·4%)	247 (4·1%)		381 (0·9%)	60 (0·1%)	76 (0·6%)		136 (0·2%)
Four times	115 (0·3%)	272 (4·5%)		387 (0·9%)	100 (0·1%)	144 (1·2%)		244 (0·3%)
**Irritable bowel syndrome**								
Present	597 (1·7%)	339 (5·6%)	<0·001	936 (2·3%)	288 (0·4%)	142 (1·1%)	<0·001	430 (0·5%)
**Iron, vit B12, or folate deficiency**								
Present	193 (0·5%)	429 (7·1%)	<0·001	622 (1·5%)	104 (0·1%)	163 (1·3%)	<0·001	267 (0·3%)
**Mouth ulcers**								
Present	331 (0·9%)	170 (2·8%)	<0·001	501 (1·2%)	142 (0·2%)	76 (0·6%)	<0·001	218 (0·2%)
**Mouth ulcers (count, 1 year)**								
Once	50 (0·1%)	42 (0·7%)	<0·001	92 (0·2%)	32 (0·0%)	19 (0·2%)	<0·001	51 (0·1%)
Twice	4 (0·0%)	9 (0·1%)		13 (0·0%)	3 (0·0%)	8 (0·1%)		11 (0·0%)
**Osteoporosis**								
Present	118 (0·3%)	145 (2·4%)	<0·001	263 (0·6%)	42 (0·1%)	62 (0·5%)	<0·001	104 (0·1%)
**Psoriasis**								
Present	722 (2·0%)	237 (3·9%)	<0·001	959 (2·3%)	290 (0·4%)	85 (0·7%)	<0·001	375 (0·4%)

	Development dataset (CPRD GOLD)				External validation dataset (CPRD Aurum)			
**Type 1 diabetes**								
Present	119 (0·3%)	126 (2·1%)	<0·001	245 (0·6%)	324 (0·4%)	150 (1·2%)	<0·001	474 (0·5%)
**Thyroid disorders**								
Present	389 (1·1%)	287 (4·8%)	<0·001	676 (1·6%)	166 (0·2%)	130 (1·0%)	<0·001	296 (0·3%)
**Weight loss**								
Present	340 (1·0%)	467 (7·7%)	<0·001	807 (2·0%)	85 (0·1%)	100 (0·8%)	<0·001	185 (0·2%)

Model selection

Table S6 Predictor selection for children, women, and men

Using a model with optimized L1 and L2 penalties performed on 200 bootstrap samples. Predictors highlighted in yellow were included in the final model. Predictors highlighted in orange were excluded because of an estimated inverse relationship with CD.

[a] Proportion of bootstrap samples in which the predictor was included in the model (non-zero coefficient).

	Children		Women		Men	
Predictor	Proportion included[a]	Median (IQR) beta coefficient	Proportion included[a]	Median (IQR) beta coefficient	Proportion included[a]	Median (IQR) beta coefficient
Abnormal liver function test results	x	x	0·615	0·032 (0, 0·175)	0·705	0·121 (0, 0·228)
ADHD	0·635	-0·117 (-0·318, 0)	0·62	0 (0, 0·361)	0·26	0 (0, 0)
Age	0·895	0·007 (0·003, 0·011)	1	-0·004 (-0·005, -0·004)	1	0·009 (0·008, 0·009)
Amenorrhoea	0·725	0·376 (0, 0·845)	0·975	-0·118 (-0·158, -0·081)	x	x
Anaemia	0·95	2·644 (2·497, 2·759)	1	1·583 (1·553, 1·617)	1	2·378 (2·318, 2·423)
Arthritis	0·865	0·93 (0·382, 1·447)	0·805	-0·083 (-0·17, -0·013)	0·425	0 (0, 0·085)
Cardiovascular disease	0·42	0 (0, 0·156)	1	0·179 (0·143, 0·225)	1	0·258 (0·219, 0·294)
Chronic liver disease	0·545	-0·033 (-0·331, 0)	0·98	0·3 (0·225, 0·386)	0·96	0·201 (0·114, 0·287)
Delayed puberty	0·875	1·261 (0·651, 1·723)	x	x	0·015	0 (0, 0)
Down’s syndrome	0·95	2·177 (1·829, 2·508)	0·875	0·899 (0·398, 1·315)	0·77	0·806 (0·081, 1·309)
Epilepsy	0·245	0 (0, 0)	0·925	0·232 (0·13, 0·319)	0·755	0·082 (0, 0·21)

	Children		Women		Men	
Predictor	Proportion included[a]	Median (IQR) beta coefficient	Proportion included[a]	Median (IQR) beta coefficient	Proportion included[a]	Median (IQR) beta coefficient
Failure to thrive	0·95	1·309 (1·087, 1·463)	0·56	0·064 (0, 1·048)	0·395	0 (0, 0·905)
Fatigue	0·95	0·506 (0·371, 0·629)	1	0·143 (0·121, 0·17)	1	0·179 (0·141, 0·215)
Fatigue (count, 1 year)	0·95	1·044 (0·883, 1·209)	1	0·365 (0·319, 0·408)	1	0·513 (0·462, 0·567)
Fatigue (count, 2 years)	0·355	0 (0, 0·084)	1	0·172 (0·145, 0·2)	0·775	0·045 (0·004, 0·082)
Fatigue (count, 10 years)	0·055	0 (0, 0)	0·2	0 (0, 0)	0·14	0 (0, 0)
First-degree relative	0·95	3·158 (3·068, 3·242)	1	2·337 (2·264, 2·421)	1	2·021 (1·93, 2·098)
Fractures	0·29	0 (0, 0·01)	0·425	0 (0, 0·016)	0·25	0 (0, 0)
Fractures (count, 1 year)	0·72	0·086 (0, 0·174)	0·965	0·118 (0·071, 0·159)	0·175	0 (0, 0)
Fractures (count, 2 years)	0·065	0 (0, 0)	0·735	0·04 (0, 0·072)	0·495	0 (0, 0·04)
Fractures (count, 10 years)	0·325	0 (0, 0·02)	0·275	0 (0, 0)	0·115	0 (0, 0)
GI symptoms	0·95	0·275 (0·221, 0·331)	1	0·331 (0·307, 0·355)	1	0·383 (0·362, 0·4)
GI symptoms (count, 1 year)	0·95	0·634 (0·589, 0·67)	1	0·506 (0·494, 0·518)	1	0·556 (0·542, 0·574)
GI symptoms (count, 2 years)	0·945	0·132 (0·098, 0·166)	1	0·118 (0·106, 0·128)	1	0·166 (0·151, 0·178)
GI symptoms (count, 10 years)	0·95	0·123 (0·096, 0·145)	1	-0·059 (-0·068, -0·05)	0·03	0 (0, 0)
Headaches and migraine	0·135	0 (0, 0)	0·245	0 (0, 0)	0·14	0 (0, 0)
Headaches and migraine (count, 1 year)	0·34	0 (0, 0·024)	0·385	0 (0, 0)	0·5	0 (0, 0·054)
Headaches and migraine (count, 2 years)	0·13	0 (0, 0)	0·355	0 (0, 0·007)	0·205	0 (0, 0)
Headaches and migraine (count, 10 years)	0·11	0 (0, 0)	0·7	-0·012 (-0·029, 0)	0·13	0 (0, 0)
Hyposplenism or functional asplenia	x	x	0·655	-0·157 (-0·477, 0)	0·505	0·001 (0, 0·378)
IgA deficiency	0·925	1·869 (0·842, 2·158)	0·83	0·739 (0·034, 1·428)	0·645	0·419 (0, 1·291)
IgA nephropathy	0	0 (0, 0)	0·4	0 (0, 0)	0·225	0 (0, 0)

	Children		Women		Men	
Predictor	Proportion included[a]	Median (IQR) beta coefficient	Proportion included[a]	Median (IQR) beta coefficient	Proportion included[a]	Median (IQR) beta coefficient
Inflammatory bowel disease	0·59	0 (0, 0·904)	0·785	0·113 (0, 0·194)	0·485	0 (0, 0·122)
Iron, Vit B12, or Folate deficiency	0·95	1·869 (1·51, 2·174)	1	1·289 (1·251, 1·333)	1	1·599 (1·525, 1·662)
Irritability	0·14	0 (0, 0)	0·91	-0·256 (-0·39, -0·134)	0·12	0 (0, 0)
Irritability (count, 1 year)	0·275	0 (0, 0)	0·39	0 (0, 0·029)	0·295	0 (0, 0)
Irritability (count, 2 years)	0·41	0 (0, 0·228)	0·52	-0·018 (-0·173, 0)	0·215	0 (0, 0)
Irritability (count, 10 years)	0·155	0 (0, 0)	0·475	0 (-0·134, 0)	0·195	0 (0, 0)
Irritable bowel syndrome	0·93	0·987 (0·607, 1·247)	1	0·485 (0·451, 0·513)	1	0·57 (0·514, 0·621)
Mood disorders	0·895	0·236 (0·124, 0·329)	0·98	-0·054 (-0·074, -0·037)	0·4	0 (0, 0·019)
Mouth ulcers	0·53	0·028 (0, 0·229)	0·675	0·037 (0, 0·109)	0·975	0·217 (0·144, 0·311)
Mouth ulcers (count, 1 year)	0·285	0 (0, 0·012)	1	0·589 (0·467, 0·696)	0·99	0·501 (0·37, 0·651)
Mouth ulcers (count, 2 years)	0·72	0·213 (0, 0·407)	0·685	0·049 (0, 0·17)	0·935	0·291 (0·163, 0·423)
Mouth ulcers (count, 10 years)	0·565	0·039 (0, 0·226)	0·68	0·046 (0, 0·105)	0·27	0 (0, 0·006)
Multiple sclerosis	x	x	0·77	-0·171 (-0·333, 0)	0·255	0 (0, 0)
Neuropathy or ataxia	0·62	-0·383 (-0·806, 0)	0·77	0·171 (0, 0·319)	0·32	0 (0, 0)
Osteoporosis	0	0 (0, 0)	1	0·969 (0·934, 1·01)	1	1·346 (1·224, 1·432)
Pancreatitis	x	x	0·685	-0·066 (-0·249, 0)	0·51	0 (-0·174, 0)
Psoriasis	0·26	0 (0, 0)	0·805	0·05 (0·002, 0·103)	1	0·241 (0·18, 0·299)
Pulmonary haemosiderosis	x	x	x	x	0·12	0 (0, 0)
Subfertility	x	x	0·845	-0·104 (-0·162, -0·033)	0·14	0 (0, 0)
Systemic lupus erythematosus	0	0 (0, 0)	0·995	0·592 (0·383, 0·751)	0	0 (0, 0)
Thyroid disorders	0·95	2·066 (1·829, 2·277)	1	0·585 (0·556, 0·613)	1	0·798 (0·737, 0·851)

	Children		Women		Men	
Predictor	Proportion included[a]	Median (IQR) beta coefficient	Proportion included[a]	Median (IQR) beta coefficient	Proportion included[a]	Median (IQR) beta coefficient
Turner syndrome	0·95	2·989 (2·679, 3·187)	0·815	0·691 (0·123, 1·287)	x	x
Type 1 diabetes	0·95	4·315 (4·175, 4·433)	1	1·286 (1·186, 1·379)	1	1·53 (1·427, 1·634)
Type 2 diabetes	0·395	0 (-0·723, 0)	1	-0·365 (-0·422, -0·314)	0·995	-0·205 (-0·249, -0·163)
Weight loss	0·95	2·28 (2·096, 2·426)	1	0·895 (0·845, 0·933)	1	1·316 (1·269, 1·388)
Sex (male)	0·95	-0·461 (-0·494, -0·427)	x	x	x	x

Model estimation

Table S7 Model coefficients and ORs with and without shrinkage applied - Children

Selected predictors	Coefficients (apparent model)	200x Bootstrap samples median (IQR) (internal validation)	Coefficients without shrinkage (apparent model)	Odds ratios after shrinkage (apparent model)	Odds ratios without shrinkage (apparent model)	Unadjusted coefficients	Unadjusted ORs
(Intercept)	-5·119	-5·127 (-5·146; -5·108)	-5·119				
Type 1 diabetes	4·153	4·182 (4·062; 4·278)	4·794	63·648	120·796	4·318	75·038
Turner syndrome	3·949	3·908 (3·715; 4·084)	11·243	51·866	76309·782	13·955	1149686·667
IgA deficiency	3·210	3·185 (2·287; 3·563)	10·770	24·789	47560·457	12·954	422523·354
First degree relatives	3·100	3·109 (3·037; 3·172)	3·361	22·196	28·808	3·167	23·736
Anaemia	2·645	2·618 (2·522; 2·751)	2·881	14·080	17·841	2·850	17·288
Down syndrome	2·429	2·428 (2·096; 2·763)	2·724	11·344	15·240	2·490	12·061
Weight loss	2·316	2·302 (2·142; 2·485)	2·563	10·135	12·972	2·811	16·627
Thyroid disorders	2·144	2·185 (2·000; 2·395)	2·361	8·536	10·601	2·742	15·518
Iron, Vit B12, or Folate deficiency	2·016	2·013 (1·704; 2·363)	2·288	7·508	9·860	2·872	17·672

Selected predictors	Coefficients (apparent model)	200x Bootstrap samples median (IQR) (internal validation)	Coefficients without shrinkage (apparent model)	Odds ratios after shrinkage (apparent model)	Odds ratios without shrinkage (apparent model)	Unadjusted coefficients	Unadjusted ORs
Delayed puberty	1·995	1·997 (1·537; 2·577)	2·464	7·353	11·756	2·997	20·025
Failure to thrive	1·382	1·398 (1·215; 1·540)	1·517	3·981	4·558	1·888	6·606
Arthritis	1·318	1·371 (0·949; 1·738)	1·525	3·737	4·596	1·725	5·613
Irritable bowel syndrome	1·127	1·135 (0·934; 1·377)	1·246	3·087	3·476	1·765	5·842
Fatigue (count 1 year)	1·111	1·090 (0·967; 1·233)	1·249	3·036	3·487	2·139	8·491
GI symptoms (count 1 year)	0·794	0·792 (0·775; 0·817)	0·854	2·213	2·348	1·023	2·782
Fatigue	0·613	0·605 (0·500; 0·698)	0·603	1·846	1·827	1·638	5·145
GI symptoms	0·582	0·584 (0·550; 0·613)	0·603	1·790	1·828	1·304	3·684
Mood disorders	0·363	0·343 (0·250; 0·448)	0·389	1·437	1·476	0·829	2·291
Age	0·011	0·011 (0·007; 0·014)	0·014	1·011	1·014	-0·007	0·993
Sex (male)	-0·477	-0·472 (-0·502; -0·447)	-0·537	0·621	0·584	-0·585	0·557

Table S8 Model coefficients and ORs with and without shrinkage applied - Women

Selected predictors	Coefficients (apparent model)	200x Bootstrap samples median (IQR)	Coefficients without shrinkage (apparent model)	Odds ratios after shrinkage (apparent model)	Odds ratios without shrinkage (apparent model)	Unadjusted coefficients	Unadjusted ORs
(Intercept)	-5·063	-5·062 (-5·080; -5·042)	-5·057				
First degree relative	2·459	2·449 (2·378; 2·517)	2·519	11·689	12·413	2·505	12·244
Anaemia	1·630	1·635 (1·605; 1·661)	1·659	5·102	5·252	1·914	6·780

Selected predictors	Coefficients (apparent model)	200x Bootstrap samples median (IQR)	Coefficients without shrinkage (apparent model)	Odds ratios after shrinkage (apparent model)	Odds ratios without shrinkage (apparent model)	Unadjusted coefficients	Unadjusted ORs
Iron, Vit B12, or Folate deficiency	1·323	1·383 (0·554; 2·113)	1·348	3·753	3·851	1·810	6·110
Type 1 diabetes	1·277	1·337 (1·293; 1·375)	1·312	3·584	3·714	1·487	4·424
Down syndrome	1·163	1·269 (1·161; 1·358)	1·256	3·198	3·512	1·124	3·077
IgA deficiency	1·127	1·170 (0·765; 1·596)	1·266	3·087	3·545	2·223	9·235
Turner syndrome	1·080	1·057 (0·422; 1·681)	1·186	2·944	3·275	1·635	5·129
Osteoporosis	1·028	1·040 (1·000; 1·077)	1·054	2·797	2·869	1·158	3·184
Weight loss	0·910	0·895 (0·848; 0·950)	0·929	2·485	2·533	1·463	4·319
Mouth ulcers (count 1 year)	0·857	0·841 (0·767; 0·907)	0·886	2·357	2·425	1·196	3·307
Systemic lupus erythematosus	0·699	0·698 (0·532; 0·856)	0·737	2·011	2·090	1·077	2·936
GI symptoms (count 1 year)	0·604	0·604 (0·594; 0·615)	0·616	1·829	1·852	0·760	2·138
Thyroid disorders	0·599	0·598 (0·563; 0·629)	0·614	1·821	1·848	0·847	2·333
Fatigue (count 1 year)	0·545	0·544 (0·518; 0·571)	0·559	1·725	1·748	0·923	2·517
Irritable bowel syndrome	0·478	0·474 (0·450; 0·505)	0·488	1·613	1·629	0·698	2·010
Chronic liver disease	0·326	0·324 (0·245; 0·383)	0·341	1·386	1·406	0·739	2·094
Epilepsy	0·258	0·252 (0·232; 0·268)	0·277	1·295	1·319	0·614	1·848

Selected predictors	Coefficients (apparent model)	200x Bootstrap samples median (IQR)	Coefficients without shrinkage (apparent model)	Odds ratios after shrinkage (apparent model)	Odds ratios without shrinkage (apparent model)	Unadjusted coefficients	Unadjusted ORs
GI symptoms	0·249	0·251 (0·173; 0·360)	0·243	1·283	1·275	1·017	2·765
Fractures (count 1 year)	0·196	0·203 (0·167; 0·241)	0·205	1·217	1·228	0·561	1·752
Cardiovascular disease	0·196	0·190 (0·139; 0·222)	0·206	1·216	1·229	0·370	1·448
Neuropathy ataxia	0·179	0·178 (0·074; 0·311)	0·203	1·196	1·225	0·703	2·020
Fatigue	0·153	0·151 (0·127; 0·178)	0·149	1·165	1·160	0·853	2·347
Inflammatory bowel disease	0·138	0·112 (0·000; 0·227)	0·153	1·148	1·165	0·952	2·591
Psoriasis	0·048	0·047 (0·000; 0·097)	0·058	1·050	1·060	0·299	1·349
Age	-0·006	-0·006 (-0·006; -0·005)	-0·006	0·994	0·994	0·001	1·001

Table S9 Model coefficients and ORs with and without shrinkage applied - Men

Selected predictors	Coefficients (apparent model)	200x Bootstrap samples median (IQR)	Coefficients without shrinkage (apparent model)	Odds ratios after shrinkage (apparent model)	Odds ratios without shrinkage (apparent model)	Unadjusted coefficients	Unadjusted ORs
(Intercept)	-5·478	-5·488 (-5·526; -5·460)	-5·481				
Anaemia	2·685	2·689 (2·632; 2·753)	2·727	14·656	15·293	3·148	23·289
First degree relatives	2·347	2·362 (2·282; 2·461)	2·395	10·456	10·969	2·210	9·116
Iron, Vit B12, Folate deficiency	1·810	1·828 (1·754; 1·917)	1·841	6·112	6·302	2·632	13·902

Selected predictors	Coefficients (apparent model)	200x Bootstrap samples median (IQR)	Coefficients without shrinkage (apparent model)	Odds ratios after shrinkage (apparent model)	Odds ratios without shrinkage (apparent model)	Unadjusted coefficients	Unadjusted ORs
Type 1 diabetes	1·746	1·749 (1·650; 1·868)	1·787	5·730	5·972	1·840	6·297
Osteoporosis	1·554	1·549 (1·433; 1·673)	1·588	4·730	4·892	1·992	7·330
Weight loss	1·490	1·489 (1·431; 1·552)	1·514	4·438	4·545	2·154	8·619
Down syndrome	1·293	1·344 (0·856; 1·813)	1·405	3·643	4·075	1·766	5·847
Mouth ulcers (count year 1)	0·934	0·919 (0·849; 0·994)	0·965	2·544	2·624	1·503	4·495
Thyroid disorders	0·910	0·913 (0·753; 1·074)	0·928	2·484	2·530	1·499	4·477
GI symptoms (count year 1)	0·787	0·789 (0·772; 0·807)	0·799	2·197	2·223	1·037	2·821
Irritable bowel syndrome	0·709	0·714 (0·651; 0·776)	0·728	2·032	2·072	1·240	3·456
Fatigue (count year 1)	0·663	0·652 (0·592; 0·714)	0·680	1·941	1·974	1·178	3·248
GI symptoms	0·448	0·442 (0·414; 0·472)	0·447	1·565	1·563	1·348	3·850
Mouth ulcers	0·412	0·401 (0·305; 0·514)	0·427	1·510	1·533	1·118	3·059
Psoriasis	0·335	0·339 (0·265; 0·401)	0·354	1·398	1·425	0·671	1·956
Chronic liver disease	0·321	0·321 (0·236; 0·396)	0·338	1·378	1·402	0·957	2·604
Epilepsy	0·259	0·290 (0·147; 0·384)	0·291	1·296	1·338	0·672	1·958
Cardiovascular disease	0·253	0·243 (0·214; 0·282)	0·257	1·288	1·293	0·975	2·651
Fatigue	0·185	0·186 (0·136; 0·233)	0·183	1·203	1·201	1·077	2·936

Selected predictors	Coefficients (apparent model)	200x Bootstrap samples median (IQR)	Coefficients without shrinkage (apparent model)	Odds ratios after shrinkage (apparent model)	Odds ratios without shrinkage (apparent model)	Unadjusted coefficients	Unadjusted ORs
Age	0·010	0·011 (0·010; 0·011)	0·011	1·010	1·011	0·023	1·023

Model performance

Table S10 Model performance

*Calibration statistics were estimated using an inflated control group to adjust for sampling frequency. FDR: first-degree relative with coeliac disease.

	**Apparent model performance**	**Internally validated model performance**	**Externally validated model performance**
**Data**	**Original data set (CPRD GOLD)**	**200x bootstrap samples of original data, median (IQR)**	**Independent data set (AURUM)**
**Children**			
**R-squared**	0·407	0·408 (0·401; 0·413)	0·065
**Brier score**	0·167	0·167 (0·165; 0·169)	0·190 / 0·156<br>Without / with FDR
***C*-statistic**	0·821	0·821 (0·818; 0·824)	0·600
**Calibration intercept***	0·147	0·161 (0·134; 0·181)	0·433 / -2·676<br>Without / with FDR
**Calibration slope***	0·964	0·986 (0·959; 1·014)	0·655
**Women**			
**R-squared**	0·237	0·248 (0·242; 0·254)	0·032
**Brier score**	0·227	0·225 (0·223; 0·227)	0·245 / 0·217<br>Without / with FDR
***C*-statistic**	0·756	0·764 (0·761; 0·767)	0·551
**Calibration intercept***	-0·161	-0·153 (-0·169; -0·143)	0·433 / -2·676<br>Without / with FDR
**Calibration slope***	0·822	0·836 (0·817; 0·855)	0·655
**Men**			
**R-squared**	0·286	0·284 (0·278; 0·291)	0·056
**Brier score**	0·122	0·124 (0·122; 0·126)	0·134 / 0·118<br>Without / with FDR
***C*-statistic**	0·798	0·796 (0·793;0·801)	0·619
**Calibration intercept***	-0·505	-0·515 (-0·534;-0·497)	0·112 / -2·250<br>Without / with FDR
**Calibration slope***	0·934	0·840 (0·817; 0·867)	0·668

Figure S1 Calibration curves model development and external validation

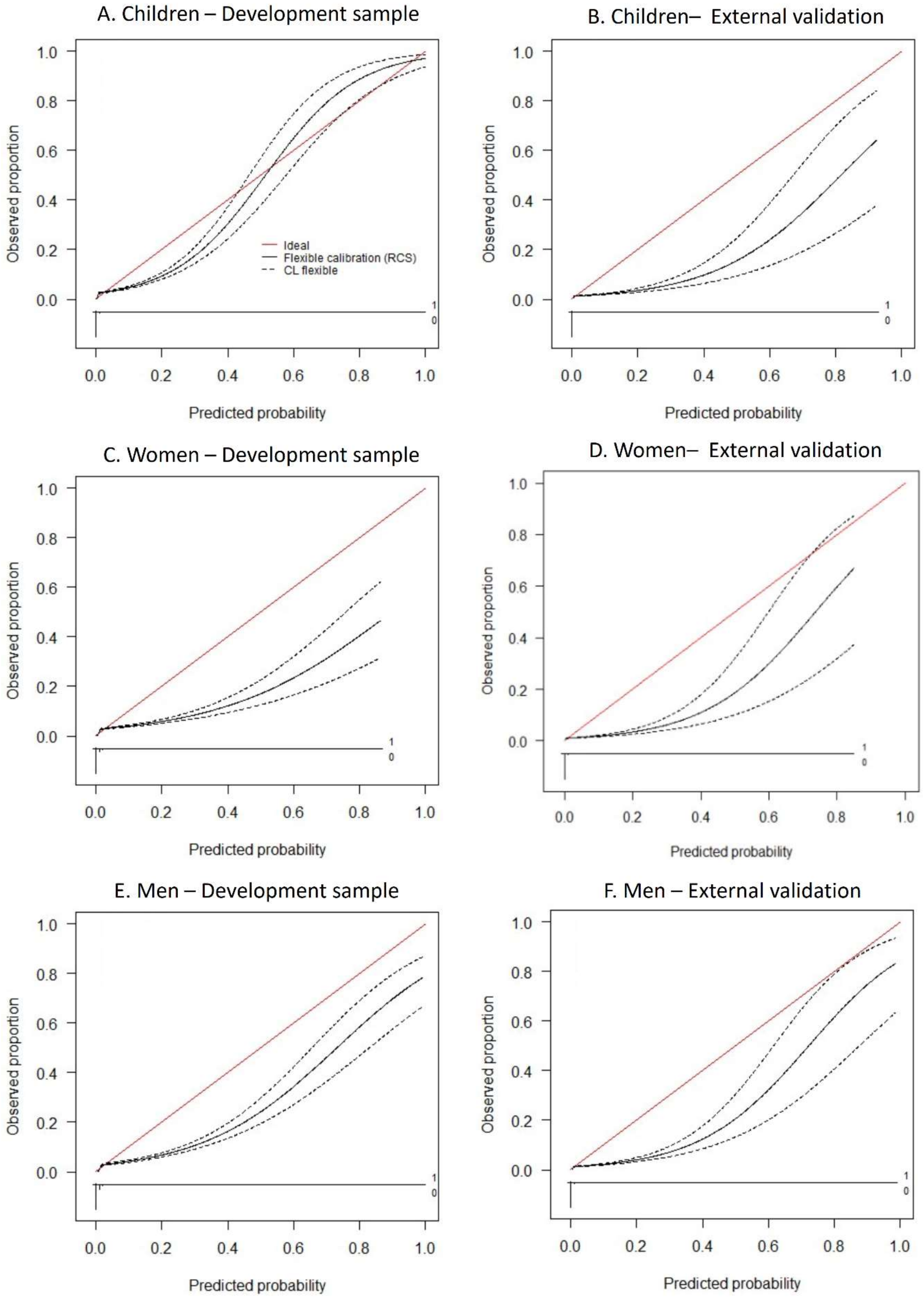

External validation

Table S11 Clinical usefulness in external validation data

In a population of 10,000 people.

Population	Threshold	TP	FP	FN	TN	Sens	Spec	PPV	NPV	% CD patients missed
Children	0	100	9900	0	0	100·0%	0%	1·0%	NA	0
	0·0038	94	9179	6	721	93·5%	7·3%	1·0%	99·1%	6·5
	0·0042	75	6297	25	3603	74·6%	36·4%	1·2%	99·3%	25·4
	0·0077	13	402	87	9498	12·6%	95·9%	3·0%	99·1%	87·4
	0·0170	10	159	90	9741	10·0%	98·4%	5·9%	99·1%	90
	0·0800	5	38	95	9862	5·2%	99·6%	12·1%	99·0%	94·8
Women	0	100	9900	0	0	100·0%	0%	1·0%	NA	0
	0·0053	32	2327	68	7574	32·2%	76·5%	1·4%	99·1%	67·8
	0·0062	14	683	86	9217	14·3%	93·1%	2·0%	99·1%	85·7
	0·0233	7	158	93	9742	7·2%	98·4%	4·5%	99·1%	92·8
	0·1070	2	20	98	9880	1·7%	99·8%	7·1%	99·0%	98·3
	0·7550	0	0	100	9900	0·0%	100·0%	6·2%	99·0%	100
Men	0	100	9900	0	0	100·0%	0%	1·0%	NA	0
	0·007	64	4623	36	5277	64·3%	53·3%	1·4%	99·3%	35·7
	0·008	42	2376	58	7524	41·6%	76·0%	1·7%	99·2%	58·4
	0·0185	11	228	89	9672	10·8%	97·7%	4·5%	99·1%	89·2
	0·0610	6	69	94	9831	6·3%	99·3%	8·2%	99·1%	93·7
	0·2820	2	10	98	9890	2·0%	99·9%	14·2%	99·0%	98

Sensitivity analyses

Table S12 Model performance after including ethnicity and deprivation as predictions

*Calibration statistics were estimated using an inflated control group to adjust for sampling frequency.

	Apparent model performance	Apparent model performance	Updated model performance
Data	Original data set (CPRD GOLD)	CPRD GOLD linked with HES	CPRD GOLD linked with HES
Children			
R-squared	0·407	0·422	0·426
Brier score	0·167	0·181	0·113
C statistic	0·821	0·824	0·824
Calibration intercept*	0·147	-0·467	-3·483
Calibration slope*	0·964	0·978	0·941
Women			
R-squared	0·237	0·272	0·276
Brier score	0·227	0·244	0·153

C statistic	0·756	0·778	0·779
Calibration intercept*	-0·161	-0·307	-3·729
Calibration slope*	0·822	0·816	0·818
Men			
R-squared	0·286	0·300	0·301
Brier score	0·122	0·153	0·113
C statistic	0·798	0·792	0·793
Calibration intercept*	-0·505	-0·768	-3·228
Calibration slope*	0·934	0·802	0·843

References

1. World Organization of Family Doctors. International Classification of Primary Care, 2nd edition (ICPC-2). 2015.

2. Holmes GKT, Muirhead A. Epidemiology of coeliac disease in a single centre in Southern Derbyshire 1958-2014. *BMJ Open Gastroenterol* 2017; **4**(1): e000137.

3. Moons KGM, Wolff RF, Riley RD, et al. PROBAST: A Tool to Assess Risk of Bias and Applicability of Prediction Model Studies: Explanation and Elaboration. *Ann Intern Med* 2019; **170**(1): W1-W33.

4. Biesheuvel CJ, Vergouwe Y, Oudega R, Hoes AW, Grobbee DE, Moons KGM. Advantages of the nested case-control design in diagnostic research. *BMC Medical Research Methodology* 2008; **8**(1): 48.

5. Moons KG. Criteria for Scientific Evaluation of Novel Markers: A Perspective. *Clinical Chemistry* 2010; **56**(4): 537-41.

6. Harrell FE. Regression Modeling Strategies. With Applications to Linear Models, Logistic and Ordinal Regression, and Survival Analysis. *Springer Series in Statistics* 2015.
