# Supplementary material for: Development and external validation of a clinical prediction model to aid coeliac disease diagnosis in primary care: An observational study
Source: eClinicalMedicine. 2022 Apr 7;46:101376. doi: 10.1016/j.eclinm.2022.101376 (PMC9011008; doi:10.1016/j.eclinm.2022.101376)
Supplement: Supplementary file 3 [file mmc3.docx]

INDEPENDENT SCIENTIFIC ADVISORY COMMITTEE (ISAC) PROTOCOL APPLICATION FORM

PART 1: APPLICATION FORM

***IMPORTANT***

**Both parts of this application must be completed in accordance with the guidance note ‘Completion of the ISAC Protocol Application Form’, which can be found on the CPRD website (**[**https://cprd.com/research-applications**](https://cprd.com/research-applications)**).**

| FOR ISAC USE ONLY | |
| --- | --- |
| **Protocol No. -** | **Submission date -** |

| GENERAL INFORMATION ABOUT THE PROPOSED RESEARCH STUDY |
| --- |
| Study Title (Max. 255 characters including spaces) Developing the optimum strategy for coeliac disease case finding in adults and children. |
| **Research Area** (place ‘X’ in all boxes that apply) |
| \| Drug Safety \|  \| Economics \| X \| \| --- \| --- \| --- \| --- \| \| Drug Utilisation \|  \| Pharmacoeconomics \|  \| \| Drug Effectiveness \|  \| Pharmacoepidemiology \|  \| \| Disease Epidemiology \| X \| Methodological \|  \| \| Health Services Delivery \| X \|  \|  \| |
| Chief Investigator  \| Title: \| Dr \| Dr \| \| --- \| --- \| --- \| \| Full name: \| Penny Whiting \| Martha Elwenspoek \| \| Job title: \| Associate Professor \| Research Associate \| \| Affiliation/organisation: \| Bristol Medical School \| ARC West, Bristol Medical School \| \| Email address: \| Penny.Whiting@bristol.ac.uk \| Martha.Elwenspoek@bristol.ac.uk \| \| CV Number (if applicable): \| 060_16S \|  \| \| Will this person be analysing the data? (Y/N) \| N \| Y \| |
| Corresponding Applicant  \| Title: \| Dr \| \| --- \| --- \| \| Full name: \| Martha Elwenspoek \| \| Job title: \| Research Associate \| \| Affiliation/organisation: \| ARC West, Bristol Medical School \| \| Email address: \| Martha.Elwenspoek@bristol.ac.uk \| \| CV Number (if applicable): \| 416_18 \| \| Will this person be analysing the data? (Y/N) \| Y \| |
| List of all investigators/collaborators  \| Title: \| Ms \| \| --- \| --- \| \| Full name: \| Joni Jackson \| \| Job title: \| Research associate \| \| Affiliation/organisation: \| Bristol Medical School \| \| Email address: \| Joni.jackson@bristol.ac.uk \| \| CV Number (if applicable): \| **970_16** \| \| Will this person be analysing the data? (Y/N) \| Y \|  \| Title: \| Ms \| \| --- \| --- \| \| Full name: \| Rachel O’Donnell \| \| Job title: \| Research associate \| \| Affiliation/organisation: \| Bristol Medical School \| \| Email address: \| bb19384@bristol.ac.uk \| \| CV Number (if applicable): \| **601_20** \| \| Will this person be analysing the data? (Y/N) \| Y \|  \| Title: \| Dr \| \| --- \| --- \| \| Full name: \| Peter Gillett \| \| Job title: \| Hon Senior Lecturer and Consultant Paediatric Gastroenterologist \| \| Affiliation/organisation: \| Royal Hospital for Sick Children, Edinburgh \| \| Email address: \| Peter.Gillett@nhs.net \| \| CV Number (if applicable): \|  \| \| Will this person be analysing the data? (Y/N) \| N \|  \| Title: \| Dr \| \| --- \| --- \| \| Full name: \| Gerry Robins \| \| Job title: \| Consultant Gastroenterologist \| \| Affiliation/organisation: \| York Teaching Hospital NHS Foundation Trust \| \| Email address: \| Gerry.Robins@york.nhs.uk \| \| CV Number (if applicable): \|  \| \| Will this person be analysing the data? (Y/N) \| N \|  \| Title: \| Dr \| \| --- \| --- \| \| Full name: \| Hazel Everitt \| \| Job title: \| Professor and GP \| \| Affiliation/organisation: \| University of Southampton \| \| Email address: \| H.A.Everitt@soton.ac.uk \| \| CV Number (if applicable): \|  \| \| Will this person be analysing the data? (Y/N) \| N \|  \| Title: \| Dr \| \| --- \| --- \| \| Full name: \| Hayley Jones \| \| Job title: \| Senior Research Fellow \| \| Affiliation/organisation: \| Bristol Medical School, University of Bristol \| \| Email address: \| Hayley.Jones@bristol.ac.uk \| \| CV Number (if applicable): \| 012_17 \| \| Will this person be analysing the data? (Y/N) \| N \|  \| Title: \| Dr \| \| --- \| --- \| \| Full name: \| Jessica Watson \| \| Job title: \| NIHR Doctoral Research Fellow and GP \| \| Affiliation/organisation: \| Bristol Medical School, University of Bristol \| \| Email address: \| Jessica.Watson@bristol.ac.uk \| \| CV Number (if applicable): \| 010_17 \| \| Will this person be analysing the data? (Y/N) \| N \|  \| Title: \| Dr \| \| --- \| --- \| \| Full name: \| Alastair Hay \| \| Job title: \| Professor and GP \| \| Affiliation/organisation: \| Bristol Medical School, University of Bristol \| \| Email address: \| Alastair.Hay@bristol.ac.uk \| \| CV Number (if applicable): \|  \| \| Will this person be analysing the data? (Y/N) \| N \|  \| Title: \| Dr \| \| --- \| --- \| \| Full name: \| Howard Thom \| \| Job title: \| Lecturer in Health Economics, Research Fellow in Statistical Modelling \| \| Affiliation/organisation: \| Bristol Medical School, University of Bristol \| \| Email address: \| howard.thom@bristol.ac.uk \| \| CV Number (if applicable): \|  \| \| Will this person be analysing the data? (Y/N) \| N \|  \| Title: \| Dr \| \| --- \| --- \| \| Full name: \| Susan Mallett \| \| Job title: \| Professor in Diagnostic and Prognostic Medical Statistics \| \| Affiliation/organisation: \| Institute of Applied Health Research, University of Birmingham \| \| Email address: \| S.Mallett@bham.ac.uk \| \| CV Number (if applicable): \|  \| \| Will this person be analysing the data? (Y/N) \| N \| \|  \|  \| \| Title: \| Ms \| \| Full name: \| Edna Keeney \| \| Job title: \| Senior Research Associate in Statistical and Health Economic Modelling \| \| Affiliation/organisation: \| Health Economics Bristol, Population Health Sciences, University of Bristol \| \| Email address: \| [Edna.keeney@bristol.ac.uk](mailto:Edna.keeney@bristol.ac.uk) \| \| CV Number (if applicable): \|  \| \| Will this person be analysing the data? (Y/N) \| Y \| |
| Experience/expertise available List below the member(s) of the research team who have experience with CPRD data.   \| **Name(s):** \| \| --- \| \| Martha Elwenspoek \| \| Penny Whiting \| \| Jessica Watson \| \| Alastair Hay \| \|  \|   List below the member(s) of the research team who have statistical expertise.   \| **Name(s):** \|  \| \| --- \| --- \| \| Martha Elwenspoek \| \| \| Penny Whiting \| \| \| Hayley Jones \| \| \| Susan Mallett \| \| \|  \| \|   List below the member(s) of the research team who have experience of handling large datasets (greater than 1 million records).   \| **Name(s):** \|  \| \| --- \| --- \| \| Jessica Watson \| \| \|  \| \| \|  \| \|   List below the member(s) of the research team, or supporting the research team, who have experience of practicing in UK primary care.   \| **Name(s):** \|  \| \| --- \| --- \| \| Jessica Watson \| \| \| Alastair Hay \| \| \| Hazel Everitt \| \| \|  \| \| |
| ACCESS TO THE DATA |
| Sponsor of the study  \| Institution/Organisation: \| University of Bristol \| \| --- \| --- \| \| Address: \| Senate House Tyndall Avenue Bristol BS8 1TH \| |
| Funding source for the study  \| Same as Sponsor? \| Yes \|  \| No \| x \|  \| \| --- \| --- \| --- \| --- \| --- \| --- \| \| Institution/Organisation: \| National Institute for Health Research (NIHR) \| \| \| \| \| \| Address: \|  \| \| \| \| \| |
| Institution conducting the research  \| Same as Sponsor? \| Yes \| x \| No \|  \|  \| \| --- \| --- \| --- \| --- \| --- \| --- \| \| Institution/Organisation: \| University of Bristol \| \| \| \| \| \| Address: \| Senate House Tyndall Avenue Bristol BS8 1TH \| \| \| \| \| |
| Data Access Arrangements Indicate with an ‘**X**’ the method that will be used to access the data for this study:   \| Study-specific Dataset Agreement \|  \| \| --- \| --- \|  \| Institutional Multi-study Licence \| X \|  \| \| --- \| --- \| --- \| \| Institution Name \| University of Bristol \| \| \| Institution Address \| Senate House Tyndall Avenue Bristol BS8 1TH \| \|   Will the dataset be extracted by CPRD?   \| Yes \|  \| No \| X \| \| --- \| --- \| --- \| --- \|   If yes, provide the reference number: |
| 1. **Data Processor(s):**  \| Processing \| X \|  \| \| --- \| --- \| --- \| \| Accessing \| X \| \| Storing \| X \| \| Processing area (UK/EEA/Worldwide) \| \| UK \| \| Organisation name \| \| University of Bristol \| \| Organisation address \| \| Senate House Tyndall Avenue Bristol BS8 1TH \| |
| INFORMATION ON DATA |
| Primary care data (place ‘X’ in all boxes that apply)  \| CPRD GOLD \| X \| CPRD Aurum \| X \| \| --- \| --- \| --- \| --- \|   **X**  Reference number (if applicable): |
| Please select any linked data or data products being requested **Patient Level Data** (place ‘**X**’ in all boxes that apply) |
| \| ONS Death Registration Data \| X \|  \| \| \| --- \| --- \| --- \| --- \| \| HES Admitted Patient Care \| X \|  \|  \| \| HES Outpatient \|  \|  \|  \| \| HES Accident and Emergency \|  \| NCRAS Cancer Registration Data \|  \| \| HES Diagnostic Imaging Dataset \|  \| NCRAS Cancer Patient Experience Survey (CPES) data \|  \| \| HES PROMS (Patient Reported Outcomes Measure) \|  \| NCRAS Systemic Anti-Cancer Treatment (SACT) data \|  \| \| CPRD Mother Baby Link \|  \| NCRAS National Radiotherapy Dataset (RTDS) data \|  \| \| Pregnancy Register \|  \| NCRAS Quality of Life Cancer Survivors Pilot (QOLP) \|  \| \| Mental Health Data Set (MHDS) \|  \| NCRAS Quality of Life Colorectal Cancer Survivors (QOLC) \|  \| |
| **Area Level Data** (place ‘**X**’ in one Practice / Patient level box that may apply)   \| **Practice level (UK)** \|  \| **Patient level (England only)** \|  \| \| --- \| --- \| --- \| --- \| \| Practice Level Index of Multiple Deprivation \|  \| Patient Level Index of Multiple Deprivation \| X \| \| Practice Level Index of Multiple Deprivation  (index other than the most recent) \|  \| Patient Level Index of Multiple Deprivation Domains \|  \| \| Practice Level Index of Multiple Deprivation Domains \|  \| Patient Level Carstairs Index for 2011 Census \|  \| \| Practice Level Carstairs Index for 2011 Census (Excluding Northern Ireland) \|  \| Patient Level Townsend Score \|  \| \| 2011 Rural-Urban Classification at LSOA level \|  \| 2011 Rural-Urban Classification at LSOA level \|  \|   Reference / Protocol number (where applicable): |
| Are you requesting linkage to a dataset not listed above?  \| Yes \|  \| No \| **X** \| \| --- \| --- \| --- \| --- \|   If yes, provide the Non-Standard Linkage reference number: |
| Does any person named in this application already have access to any of these data in a patient identifiable form, or associated with an identifiable patient index?  \| Yes \|  \| No \| **X** \| \| --- \| --- \| --- \| --- \|   If yes, provide further details: |
| VALIDATION/VERIFICATION |
| Does this protocol describe an observational study using purely CPRD data?  \| Yes \|  \| No \| **X** \| \| --- \| --- \| --- \| --- \| |
| Does this protocol involve requesting any additional information from GPs, or contact with patients?  \| Yes \|  \| No \| **X** \| \| --- \| --- \| --- \| --- \|   If yes, provide the reference number: |

PART 2: PROTOCOL INFORMATION

| **Applicants must complete all sections listed below**  **Applications with sections marked ‘Not applicable’ without justification will be returned as invalid** |
| --- |
| Study Title (Max. 255 characters, including spaces) Developing the optimum strategy for coeliac disease case finding in adults and children. |
| Lay Summary (Max. 250 words) Coeliac disease (CD) is an autoimmune disease affecting the digestive system that causes an adverse reaction to gluten. Untreated CD can increase the risk of anaemia, osteoporosis, cancer, and infertility. Around 1 in 100 people in the UK have CD, although many are not diagnosed. There is a need for clear, evidence-based guidance for identifying adults and children with CD to speed up the diagnostic process and improve patient outcomes.  We want to establish who should be tested for CD, what tests should be offered, and whether a biopsy is necessary in all patients. We also want to investigate the cost-effectiveness of different strategies. We will do this by reviewing existing studies on tests for CD and studies on what symptoms, signs, or other factors are associated with CD; by analysing routinely collected data to see which factors may predict CD; by working with patients to help us work out which strategies are best for patients; and by economic evaluation of each strategy to find the most cost-effective strategy. |
| Technical Summary (Max. 300 words) Coeliac disease (CD) is an immune-mediated disorder, triggered by the protein gluten, estimated to affect 1% of the UK population. Some patients with CD may be asymptomatic, others present with non-specific symptoms, making diagnosis difficult; only 24% are thought to be diagnosed. Untreated CD may lead to malnutrition, anaemia, osteoporosis, infertility in women, lymphoma, and small bowel cancer.  Guidelines recommend that adults and children “at high risk” of CD should be offered testing. However, it is not clear which groups are at sufficiently high risk to justify routine testing, which symptoms should prompt testing, which tests should be offered, and whether confirmatory biopsy is necessary.  We are currently conducting a systematic review on the accuracy of diagnostic indicators (e.g. risk factors and symptoms) for CD. Diagnostic indicators associated with an increased risk of CD will be selected to inform the development of prediction models using CPRD GOLD data. We will fit logistic regression models with CD as the outcome and multiple predictors. From the results, we will estimate the probability of CD diagnosis for each combination of diagnostic indicators available. We will validate the model developed in CPRD GOLD using bootstrapping (internal validation) and in CPRD Aurum (external validation). We will also validate any existing prediction model identified by the systematic review in CPRD Aurum.  Finally, the cost-effectiveness of CD testing of patients with pre-test probabilities of CD above certain thresholds will be evaluated with a long-term economic model. We will use CPRD Aurum linked to HES to estimate the risks of adverse outcomes in patients with CD (such as lymphoma or infertility) and the risk of adverse events, including death, associated with biopsy. These estimates will directly inform the economic model. |
| Outcomes to be Measured *Prediction modelling (case control) and validation of existing prediction models:* the primary outcome is diagnosed CD (see Appendix A for Read codes).  *Estimating risk of adverse long-term outcomes associated with CD and adverse events associated with biopsy (cohort):* key outcomes are all-cause mortality; any malignancy; Hodgkin-lymphoma; non-Hodgkin-lymphoma; small intestinal cancer; colon cancer; splenic hypofunction; osteoporosis; iron deficiency anaemia; vitamin B12 and folate deficiency anaemia; pregnancy-related complications, including unexplained infertility, recurrent miscarriage or intrauterine growth restriction (see Appendix B for ICD-10 codes); and perforation, infection, bleeding, sepsis, myocardial infarction, and death associated with biopsy (see Appendix C for ICD-10 and Read codes). |
| Objectives, Specific Aims and Rationale **Rationale**: There is a need for clear, evidence-based guidance on who should be offered testing for CD to speed up the diagnostic process and improve patient outcomes.  **General objective**: To determine the cost-effectiveness of active case-finding for CD in primary care.  **Specific aims** (aims in *italic* do not require CPRD data):   1. *Systematic review to identify important diagnostic indicators that are associated with a higher risk of having CD and to find existing prediction models for CD (underway)* 2. Develop a prediction model for diagnosed CD using CPRD Gold data.    1. Develop separate models for adults and children using the diagnostic indicators identified in the systematic review    2. Internal validation of the model developed in CPRD Gold, using bootstrapping. 3. Validate prediction models using CPRD Aurum data    1. External validation of the model developed in 2 in CPRD Aurum.    2. Validate any prediction models identified by the systematic review on diagnostic indicators 4. Economic modelling to identify the cost-effectiveness of different active case-finding strategies.    1. Estimate the risks of adverse outcomes in patients with CD and those without CD using CPRD data, which will feed into the economic model.    2. Estimate the risk of adverse events, including death, associated with biopsy using CPRD data, which will feed into the economic model.    3. *Development of economic model.* |
| Study Background Coeliac disease (CD) is an autoimmune disorder, triggered by the protein gluten, found in wheat, rye and barley.(1) Some patients with CD may be asymptomatic, others present with non-specific symptoms including gastrointestinal symptoms (e.g. diarrhoea, bloating, gassiness, constipation, vomiting and abdominal pain), fatigue, and unexplained weight loss. CD is estimated to affect around 1% of people in the UK,(2) however only 24% of those with the condition are thought to be diagnosed.(3) Treatment for CD is lifetime adherence to a gluten free diet, which can be difficult and restrictive, significantly impacting quality of life. Thus, the accuracy of the diagnostic strategy for the condition is particularly important. If CD is not diagnosed promptly and the condition remains untreated, damage may be sustained to the surface of the small intestine and difficulty absorbing nutrients may lead to malnutrition, anaemia and/or osteoporosis.(3) In the long-term, untreated CD may lead to a higher risk of serious complications, such as infertility in women, lymphoma and small bowel cancer.(4, 5)  Within the current diagnostic pathway for CD, adults and children “at high risk” of CD should be offered testing. However, there is a lack of consensus regarding who should be tested and whether certain patient groups are at sufficiently high risk to justify routine testing. It is also not clear what symptoms are suggestive of CD and should prompt testing. NICE guidelines recommend that patients are first tested for immunoglobulin A (IgA) and IgA anti-tissue transglutaminase (tTG). Depending on the results, and whether adults or children are being tested, further testing, including intestinal biopsy may be required. The latter is invasive, expensive, potentially distressing, and burdensome, with risks of complications particularly for children, who require general anaesthesia to undergo the procedure. Biopsy is recommended to confirm a diagnosis of CD in all adults with positive serological test results, regardless of how strongly indicative their results are of CD. Patients must eat gluten daily in the six weeks  prior to any serological testing or biopsy for the result to be reliable.  Why is this research important?  Determining the cost-effectiveness of active case finding in different at-risk populations will help commissioners, clinicians and patients make evidence-based decisions on whether active case finding for CD is an appropriate cost-effective activity to undertake in primary care. There is evidence that CD is under diagnosed in both children and adults.(2, 6) Appropriate identification and treatment of CD can have significant benefits for patients in terms of  symptoms, quality of life, and long-term health outcomes, as well as reducing healthcare and societal economic costs.(7, 8) Different guidelines recommend different diagnostic pathways showing a lack of evidence and consensus on which groups may benefit from active case finding, the best method of doing this and the costs, benefits and potential harms. A rigorously undertaken high-quality evidence synthesis including health economic modelling will provide a robust summary of the current evidence base and highlight whether there is sufficient evidence to suggest an optimum strategy or whether further research is needed. |
| Study Type This project is primarily an exploratory study. |
| Study Design For the *diagnostic prediction model,* we will use a nested case-control design where CD is treated as the outcome.  For the *economic models*, we will use a comparative cohort design to estimate the risk of certain outcomes among people with CD and in a matched control group without coeliac disease. In this case, CD is treated as an exposure and the adverse outcomes as outcomes. When estimating the risks of adverse outcomes associated with biopsies, the biopsy is treated as the exposure.  However, both studies will use the same population of CD patients with matched controls. |
| Feasibility counts There are currently (April 2020) 19,933 patients with a CD diagnostic code in CPRD Gold and 44,772 in CPRD Aurum (see Appendix A for code lists) from GP practices that were up-to-standard since at least 12 months prior to diagnosis. Each case will be matched to four controls. This will give us a total sample size of ca. 100,000 patients in CPRD gold and 225,000 in CPRD Aurum. |
| Sample size considerations We have made the sample size considerations based on the prediction model, as this is the primary aim of this project. We expect to identify less than 100 diagnostic indicators in the systematic review and will only use diagnostic indicators that are associated with an increased risk of CD (i.e. a risk higher than in the general population). Although there is lack of consensus regarding the maximum number of events per variable (EPV), there is generally consensus that less than 10 leads to bias. With 20,000 ‘events’ (coeliac patients) in the CPRD database, we would be able to investigate a maximum of 500 predictor variables per age group (adults/children) and sex (male/female). |
| Planned use of linked data (if applicable): *Prediction modelling (case control):* no linkages.  *Adverse outcomes (cohort):* linkages to ONS, because ONS is the most reliable source for mortality data, which is one of the outcomes for the economic modelling; to HES Admitted Patient Care, to measure outcomes that are recorded in secondary care, such as lymphoma or biopsy adverse events; and to IMD2015 to include social deprivation as a potential confounding factor. |
| Definition of the Study population Target population: only permanently registered acceptable patients will be included and only up-to-standard follow-up will be considered. Patients from GP practices that were up-to-standard for at least 12 months prior to diagnosis will be included.  Follow-up period: The study start is the latest of the start of linked data, the date of patient registration with the practice and the ‘UTS date’ of that practice. The study end is the earliest of the last date for linked primary care data, the date of patient transfer-out from practice, the date of patient’s death (according to CPRD death date) or the last date of data collection from that practice.  Cases are defined as individuals with one or more Read codes related to CD (see appendix A) as previously described (data: clinical).(3) We will include patients with a diagnosis for dermatitis herpetiformis (DH), but not with DH alone (see Appendix A for full details). We will assign a date of diagnosis for each patient corresponding to the date of their first record of CD (for patients with more than one CD code, the earliest will be considered as the date of disease diagnosis). |
| Selection of comparison group(s) or controls All remaining patients without any of the CD codes are potential controls. From these, we will exclude patients with any record of gluten-free prescriptions (data: therapy), DH or gluten sensitivity diagnosis (data: clinical) using previously defined methodology.(3, 9, 10) This will reduce the risk of including undiagnosed CD patients in the control group.  Cases and controls will be matched 1:4 on age group (adult vs child), GP practice, availability of HES, ONS, and IMD2015 linkage. Controls will inherit the index date of the cases and will have a follow-up at least as long as the case. We will not match by age or sex as our analysis includes identification of incremental value of diagnostic models including sex and age and matching of covariates is problematic for this research aim including incremental value of diagnostic (classification) models. (11, 12) We will match by age group (adult vs child) to ensure there are sufficient child controls to allow an efficient study design. |
| Exposures, Outcomes and Covariates *Prediction modelling (case control):*  Predictors: diagnostic indicators identified in the systematic review, which are recorded prior to the diagnosis date (objective 2). Outcomes: record of a code for CD as listed in appendix A.1 (case) or absence of a code listed in appendix A.2 (control) identified in the primary care clinical records.  *Estimating risk of adverse outcomes (cohort):*  Exposure: record of a code for CD as listed in appendix A identified in the primary care clinical records for the long-term outcomes associated with CD and a record of duodenal biopsy as listed in appendix C. Outcomes: record of an ICD-10 code of adverse outcomes associated with CD and duodenal biopsy procedures as listed in appendix B and C. Covariates: age, sex, socioeconomic position (IMD2015 deciles). |
| Data/ Statistical Analysis *Prediction modelling (case control):*  We will fit conditional logistic regression models with CD as the outcome and multiple explanatory factors, including interaction terms where appropriate. From the results, we will produce estimates of the probability of CD diagnosis for each combination of diagnostic indicators available. We will estimate model performance using measures of both discrimination (ability of the model to distinguish between those with and without CD) and calibration (agreement between predictions and observed outcomes).(13) We will internally validate the model using bootstrapping methods to adjust estimates of model performance for overfitting and optimism.(13) We will use CPRD Aurum to externally validate the model.  *Estimating risk of adverse outcomes (cohort):*  We will estimate risks per adverse outcome using multilevel logistic regression models with a random intercept at the GP level. It is reasonable to assume that CD patients cluster by GP practice, because the awareness of CD and the propensity of testing for CD will differ per GP practice. GP practices with higher awareness of CD are more likely to diagnose patients with less severe symptoms and therefore age of diagnosis may be lower. We will deal with multiple testing by cautious interpretation of the estimates.  *Sensitivity analysis*  We will include a sensitivity analysis restricting to patients diagnosed after 1997, because in this year IgA tTG tests were first developed, which are now the preferred serological test for screening for CD. |
| Plan for addressing confounding Confounding is not relevant for the prediction model. For estimating the risk of adverse outcomes, potential confounders will be identified based on our systematic review by considering whether factors associated with CD are also likely to be associated with the outcome of interest (e.g. lymphoma) but not on the causal pathway between the two. |
| Plans for addressing missing data If diagnostic indicators (i.e. outcomes) are partially missing, missing values will be imputed using multiple imputation, assuming values are missing at random conditional on observed covariates. |
| Patient or user group involvement Patient representatives have been involved in the development of this research proposal. We have planned collaborative work with patients to identify diagnostic certainty for testing, starting treatment, and referral for biopsy, which will inform the economic model. Towards the end of the project, we will convene an implementation panel including patient representatives to advise on the optimum strategy for enhanced dissemination. We will discuss findings with the charity Coeliac UK to help with dissemination to patients. |
| Plans for disseminating and communicating study results, including the presence or absence of any restrictions on the extent and timing of publication Results will be reported in peer-reviewed journals (following TRIPOD reporting guidelines for prediction model studies and STROBE reporting guidelines for observational studies), academic, and public presentations and social media. See R. for dissemination plan to patients and wider audiences.  **Conflict of interest statement:** None |
| Limitations of the study design, data sources, and analytic methods A limitation with using routine data such as CPRD to explore questions relating to the diagnosis of CD is that CD is underdiagnosed – only an estimated 1 in 4 patients who have CD are diagnosed with the condition. This means that 3 in every four patients who actually have CD are likely to be incorrectly classified as not having CD. This is likely to bias any analysis looking at the predictive value of signs, symptoms and other risk factors for CD in favour of signs/symptoms/risk factors more commonly associated with CD as these are the patients who are most likely to have been tested. We will mitigate this risk by excluding individuals on a GFD or with a diagnosis of DH from the control group. |
| References 1. Kelly C, Leffler D. Coeliac disease. BMJ Best Practice. 2018.  2. Bingley PJ, Williams AJ, Norcross AJ, Unsworth DJ, Lock RJ, Ness AR, et al. Undiagnosed coeliac disease at age seven: population based prospective birth cohort study. BMJ. 2004;328(7435):322-3.  3. West J, Fleming KM, Tata LJ, Card TR, Crooks CJ. Incidence and prevalence of celiac disease and dermatitis herpetiformis in the UK over two decades: population-based study. Am J Gastroenterol. 2014;109(5):757-68.  4. Green PH, Jabri B. Coeliac disease. Lancet. 2003;362(9381):383-91.  5. Green PH. Mortality in celiac disease, intestinal inflammation, and gluten sensitivity. JAMA. 2009;302(11):1225-6.  6. Ravikumara M, Nootigattu VK, Sandhu BK. Ninety percent of celiac disease is being missed. J Pediatr Gastroenterol Nutr. 2007;45(4):497-9.  7. Gray AM, Papanicolas IN. Impact of symptoms on quality of life before and after diagnosis of coeliac disease: results from a UK population survey. BMC Health Serv Res. 2010;10:105.  8. Mearns ES, Taylor A, Boulanger T, Craig KJ, Gerber M, Leffler DA, et al. Systematic Literature Review of the Economic Burden of Celiac Disease. Pharmacoeconomics. 2018.  9. Abdul Sultan A, Crooks CJ, Card T, Tata LJ, Fleming KM, West J. Causes of death in people with coeliac disease in England compared with the general population: a competing risk analysis. Gut. 2015;64(8):1220-6.  10. Violato M, Gray A, Papanicolas I, Ouellet M. Resource use and costs associated with Coeliac disease before and after diagnosis in 3,646 cases: Results of a UK primary care database analysis. PLoS ONE. 2012;7(7):e41308.  11. Janes H, Pepe MS. Matching in studies of classification accuracy: implications for analysis, efficiency, and assessment of incremental value. Biometrics. 2008;64(1):1-9.  12. Janes H, Pepe MS. Adjusting for covariate effects on classification accuracy using the covariate-adjusted receiver operating characteristic curve. Biometrika. 2009;96(2):371-82.  13. Moons KGM, Wolff RF, Riley RD, Whiting PF, Westwood M, Collins GS, et al. PROBAST: A Tool to Assess Risk of Bias and Applicability of Prediction Model Studies: Explanation and Elaboration. Ann Intern Med. 2019;170(1):W1-W33. |

| List of Appendices  1. Code list for CD cases and controls 2. Long-term adverse outcomes CD 3. Adverse events associated with biopsy |
| --- |
| **AMENDMENT – 29/03/2021**  **Section C: Technical summary**  We will also use CPRD GOLD data to quantify medical costs associated with a diagnosis of coeliac disease. These estimates will directly inform the economic model.  **Section D: Outcomes to be measured**  *Estimating medical costs associated with CD*: key outcomes are number of primary care consultations, tests, referrals to out-patient hospital care and prescriptions. Section E: Specific aims  - Quantify the volume, type and costs of healthcare resources used by individuals diagnosed with CD, up to ten years before and after diagnosis, and estimate medical costs associated with CD, which will feed into the economic model.  Section F: Study background A key input to the health economic modelling is the health care use and costs associated with a diagnosis of coeliac disease. A previous study by Violato et al used a cohort of 3,646 CD cases and 32,973 matched controls, extracted from the General Practice Research Database (GPRD) over the period 1987–2005 to evaluate the impact of diagnosis on the average resource use and costs of cases and to assess direct healthcare costs due to CD by comparing average resource use and costs incurred by cases vs. controls.(10)  **Section H: Study design**  To quantify the volume, type, and costs of healthcare resources used by individuals diagnosed with CD we will use the same cohort of people with CD and matched control group without CD. This updates the Violato et al analysis with a more recent cohort of patients and reflecting current costs associated with healthcare use.(10)  **Section N: Exposures, covariates, and outcomes**  Medical costs (cohort):  Exposure: record of a code for CD as listed in appendix A identified in the primary care clinical records for the long-term outcomes associated with CD, Outcomes: primary care consultations, tests, referrals to out-patient hospital care and prescriptions.  **Section O: Data/statistical analysis**  Medical costs (cohort):  To quantify healthcare resource use, we will analyse the 10-year period prior to and following index date. The full 20-year period will be considered even if data on healthcare utilisation and costs for a case or control is available for less than 20 years, providing that data is available for at least some of the matched sets of cases and controls. As such, resource use and costs may be calculated on a number of cases/controls that is variable each year over the period of the study.  To evaluate the impact of diagnosis on the average resource use and costs incurred by each patient, average annual amounts of healthcare resource use and associated costs will be calculated and compared for the identified CD cohort in the 10 years prior to and following index date. Similar analyses will also be performed in the control cohort. Analyses will be stratified by resource use and cost categories of interest, and by age group at index date (total costs only).  The direct healthcare costs due to CD will be estimated by comparing the average annual amounts of healthcare resources uses and costs incurred by the CD cohort with the amounts incurred by the matched control cohort before and after index date. These analyses will also be stratified by resource use and cost category of interest, and by age group at index date (total costs only).  Consultations, referrals, tests, and prescriptions will be treated as continuous variables and reported as number of events per patient per year. Resource use rates and mean costs before and after index date will be reported will their standard errors. Rates and mean differences in observed resource use and costs between the two time periods will be reported alongside 95% confidence intervals (CI). Statistical differences in mean estimates will be evaluated using Student’s two-sided t-test. Unpaired t-tests will be used to conform to the decision not to restrict analyses to matched sets for which the ratio of cases to controls was consistently 1:4 over the time of the study. |
